# Dual ancestries and ecologies of the Late Glacial Palaeolithic in Britain

**DOI:** 10.1038/s41559-022-01883-z

**Published:** 2022-10-24

**Authors:** Sophy Charlton, Selina Brace, Mateja Hajdinjak, Rebecca Kearney, Thomas Booth, Hazel Reade, Jennifer A. Tripp, Kerry L. Sayle, Sonja B. Grimm, Silvia M. Bello, Elizabeth A. Walker, Alexandre Gilardet, Philip East, Isabelle Glocke, Greger Larson, Tom Higham, Chris Stringer, Pontus Skoglund, Ian Barnes, Rhiannon E. Stevens

**Affiliations:** 1 PalaeoBARN, School of Archaeology, University of Oxford, Oxford, UK; 2 The Natural History Museum, London, UK; 3 Ancient Genomics Laboratory, The Francis Crick Institute, London, UK; 4 UCL Institute of Archaeology, London, UK; 5 GFZ German Research Centre for Geosciences, Potsdam, Germany; 6 Department of Chemistry, University of San Francisco, San Francisco, CA USA; 7 Scottish Universities Environmental Research Centre, East Kilbride, UK; 8 Schleswig-Holstein State Museums Foundation Schloss Gottorf, Centre for Baltic and Scandinavian Archaeology (ZBSA), Schleswig, Germany; 9 Department of History & Archaeology, Amgueddfa Cymru—National Museum Wales, Cardiff, UK; 10 Human Evolution and Archaeological Sciences Forschungsverbund, University of Vienna, Vienna, Austria; 11 Department of Evolutionary Anthropology, University of Vienna, Vienna, Austria

**Keywords:** Archaeology, Population genetics, Palaeoecology

## Abstract

Genetic investigations of Upper Palaeolithic Europe have revealed a complex and transformative history of human population movements and ancestries, with evidence of several instances of genetic change across the European continent in the period following the Last Glacial Maximum (LGM). Concurrent with these genetic shifts, the post-LGM period is characterized by a series of significant climatic changes, population expansions and cultural diversification. Britain lies at the extreme northwest corner of post-LGM expansion and its earliest Late Glacial human occupation remains unclear. Here we present genetic data from Palaeolithic human individuals in the United Kingdom and the oldest human DNA thus far obtained from Britain or Ireland. We determine that a Late Upper Palaeolithic individual from Gough's Cave probably traced all its ancestry to Magdalenian-associated individuals closely related to those from sites such as El Mirón Cave, Spain, and Troisième Caverne in Goyet, Belgium. However, an individual from Kendrick's Cave shows no evidence of having ancestry related to the Gough’s Cave individual. Instead, the Kendrick’s Cave individual traces its ancestry to groups who expanded across Europe during the Late Glacial and are represented at sites such as Villabruna, Italy. Furthermore, the individuals differ not only in their genetic ancestry profiles but also in their mortuary practices and their diets and ecologies, as evidenced through stable isotope analyses. This finding mirrors patterns of dual genetic ancestry and admixture previously detected in Iberia but may suggest a more drastic genetic turnover in northwestern Europe than in the southwest.

## Abstract (web summary)

The authors report genetic, archaeological and stable isotopic data from two late Palaeolithic individuals in Britain, from Gough's Cave and Kendrick's Cave. The individuals differ not only in their ancestry but also their diets, ecologies and mortuary practices, revealing diverse origins and lifeways among inhabitants of late Pleistocene Britain.

## Main

The climatic warming that occurred after the Last Glacial Maximum (LGM) was critical in the development of human societies and dramatically altered the distribution of faunal and floral communities in Europe. Landscapes that were uninhabited at the LGM were recolonized during the Late Glacial and the distribution and density of human populations changed markedly, alongside the emergence of substantial cultural diversification. As such, many studies have focused on relationships between population expansion, environmental change and cultural diversity in Europe in the post-LGM period (for example, refs. ^1–5^). However, details of human postglacial recolonization of Europe remain unclear due to the complex history of prehistoric migrations across the continent and the relative paucity of human remains dating to this period.

In recent years, advances in sequencing technologies, combined with improved laboratory methods and bioinformatic workflows, have opened up the possibility to generate and analyse the genetic signatures of Late Pleistocene European populations. To date, a number of studies have explored the genetic makeup of the earliest modern humans in Europe, before the emergence of agriculture. These studies have revealed numerous instances of genetic shifts indicative of population expansions^6–9^. One of the most notable examples occurred during the Late Glacial, between the end of the LGM (~23,400 calibrated years before present (cal. bp)) and the start of the Holocene epoch (~11,700 cal. bp). This shift is reflected in the ancestries associated with the ~15,090-year-old (IntCal20) Goyet Q2 individual, Belgium, and the ~14,010-year-old (IntCal20) Villabruna individual, Italy, in post-LGM Europe. We use these individuals as shorthand for the ancestries associated with them throughout the text. ‘Goyet Q2’ ancestry^9^, which has previously been defined by the ~18,770-year-old (IntCal20) ‘El Mirón’ individual^8^ from Spain, has been identified in individuals associated with the Magdalenian culture, dating from ~20,500 to 14,000 cal. bp. This Goyet Q2/El Mirón ancestry has been suggested to represent a post-LGM expansion from southwestern European glacial refugia^8^.

The ‘Villabruna’ ancestry, also broadly known as Western hunter gatherers or WHG, consists of individuals dated from ~14,000 to 7,000 cal. bp associated with Epigravettian, Azilian/Federmesser, Epipalaeolithic and Mesolithic cultures^8^. The Villabruna ancestry is also associated with the observation that from ~14,000 cal. bp, all European individuals show some level of genetic affinity to present-day Near Eastern populations^8^. The expansion in the geographic distribution of this ancestry also correlates with a period of rapid climate warming of the Late Glacial Interstadial (considered broadly equivalent to the onset of Greenland Interstadial 1 (GI-1), ~14,650 cal. bp) as well as cultural transitions from the Magdalenian/Late Upper Palaeolithic to the Azilian/Federmesser-Gruppen/Final Palaeolithic and has therefore been suggested to represent the movement of people into northwestern Europe after the LGM^8^.

Interestingly, however, individuals with a mixture of Goyet Q2 and Villabruna ancestry appear in southern Europe from at least ~18,700 cal. bp—with the individual from El Mirón^8^ being the earliest identified thus far. The presence of individuals with admixed Goyet Q2 and Villabruna ancestry in southern Europe from the LGM onwards raises questions related to the fragmentation of populations into isolated refugia during the last Ice Age^1,10–12^. It appears that both cultural and gene flow continued across the continent—although the nature of these processes and the mechanisms involved remain unclear. However, the presence of individuals with un-admixed Goyet Q2 ancestry in northern Europe until ~14,000 cal. bp (ref. ^9^) also suggests some degree of sustained isolation throughout the LGM and into the Late Glacial. There is evidence of populations living in ice-marginal environments within northern Europe at the LGM and of long-distance movement of people from east to west north of the Alps, which has also been linked to the expansion of Magdalenian cultural groups^13–15^. This evidence raises suggestions of Magdalenian populations with Goyet Q2 ancestry—who appear to have been cold-adapted hunter gatherers—retreating to northern Europe, perhaps due to climatic warming and the movement of prey species such as reindeer and horse. Conversely, more southerly regions such as northern Spain and Italy, where temperate prey species such as red deer persisted throughout the LGM and Late Glacial, may have provided greater ecological opportunities for population admixture^16^.

Britain lies at the extreme northwest corner of the post-LGM expansion. With approximately two-thirds of the landmass covered by ice at the LGM and rapid deglaciation thereafter^17^, substantial ecological and environmental change took place in the post-LGM landscape. As such, Britain offers a unique environmental context through which Late Upper Palaeolithic populations can be considered. By ~19,000 cal. bp the British–Irish Ice Sheet was undergoing widespread melt and by ~16,000 cal. bp ice was absent from virtually all of England and Wales^17^. Reindeer were present in southwest England by ~17,000 cal. bp (ref. ^18^) and habitats were dominated by open steppe–tundra vegetation^19,20^. However, detailed consideration of Late Upper Palaeolithic sites in the United Kingdom and a series of radiocarbon dating programmes suggest that there is no evidence for post-LGM human recolonization of southwestern Britain before ~15,500 cal. bp (ref. ^21^). As such, some regions of Britain were colonized before the rapid climate warming at the start of the Late Glacial Interstadial (~14,650 cal. bp). Accelerator mass spectrometry (AMS) dating indicates that Britain was probably recolonized at a slightly later date than adjacent regions such as the Paris Basin and the Belgian Ardennes^21,22^—thereby suggesting an expansion of people across the European continent^1^. Interestingly, the British Magdalenian (known locally as the Creswellian) appears to be very similar (both in terms of chronology and cultural expression/typology) to the Classic Hamburgian, found in the northern Netherlands and the lowlands of northern Germany and Poland^22,23^. However, understanding the expansion of post-LGM populations into and within the British Isles is hindered by a relative paucity of preserved archaeological remains suitable for dating^22^. As such, the exact nature of human occupation of Late Upper Palaeolithic Britain remains unclear and we have relatively little knowledge of the earliest postglacial populations in Britain.

Whilst the genetics of Mesolithic, Neolithic and Bronze Age individuals from Britain have recently been explored^24,25^, no genetic data have yet been generated for British Palaeolithic individuals, due in part to the scarcity of human skeletal material available from Late Pleistocene Britain. To date, modern human skeletal remains have been recovered from only six Upper Palaeolithic sites^26–30^. Nonetheless, these rare samples are crucial for our understanding of human populations across post-LGM Europe due to Britain’s location on the most northwesterly fringe of the European continent. Mesolithic British populations have been identified genetically as WHGs (Villabruna ancestry), indicating that this genetic ancestry spread to the most northwesterly area of early Holocene Europe by at least ~10,500 cal. bp (ref. ^25^). What remains unclear, however, is when this ancestry first arrived in Britain and, additionally, what the genetic ancestry of Palaeolithic populations in Britain may have been. Given the previous association of Goyet Q2 ancestry with Magdalenian cultures across Europe and the similarities between the Creswellian and the Classic Hamburgian cultures, it could be hypothesized that British Late Upper Palaeolithic populations would also fall within the Goyet Q2 genetic cluster. To address these questions and expand our knowledge of the genetic makeup of Europe after the LGM, we investigate here the genetic characteristics of Late Upper Palaeolithic Britain through ancient DNA analyses of human remains from two archaeological sites in England and Wales.

## Materials

**Fig. 1:**
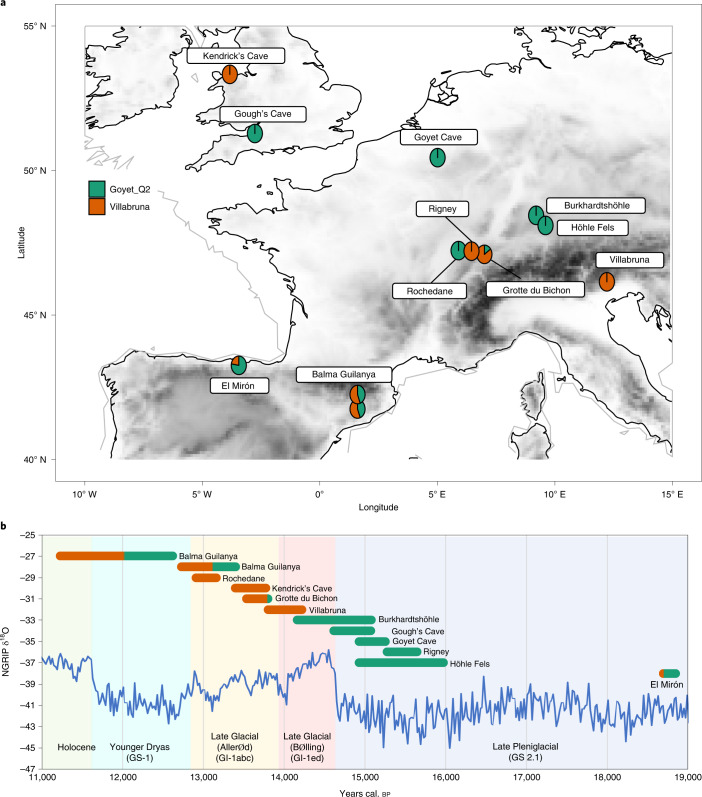
Location, genetic ancestry and AMS date of individuals discussed within the text. **a**, Map indicating the location of Pleistocene sites discussed within the text and the genetic ancestry of individuals analysed from them. **b**, NGRIP ice core δ^18^O values and INTIMATE event stratigraphy (North Greenland Ice Core Project members 2004; ref. ^42^), genetic ancestry and date of individuals (IntCal20, 95% confidence interval of calibrated radiocarbon dates for directly dated humans). The Gough’s Cave and Höhle Fels specimens are not directly dated and therefore for Höhle Fels specimen the age range shown is the 95% confidence interval of calibrated radiocarbon dates from bones recovered from the same area/context. For the Gough’s Cave specimen, the age range shown is the Bayesian modelled site occupation start and end dates based on AMS dating of the human remains and humanly modified faunal remains (Supplementary Table 7).

Among two of the most well-known sites from the British Late Upper Palaeolithic are Gough’s Cave and Kendrick’s Cave. The site of Gough’s Cave is part of a large cave system situated in Cheddar Gorge in Somerset, southwest England (Fig. 1). It is particularly well-known due to its lithic and faunal assemblages being amongst the largest of any British Palaeolithic cave site investigated thus far^21,22^. The Gough’s Cave lithic assemblage is of mixed origin, containing both late Magdalenian and early Federmesser-Gruppen technologies and the recorded start of occupation at the site coincides with the beginning of the Interstadial period (GI-1e), when temperatures rapidly increased^21,22^. As well as the Mesolithic-dated ‘Cheddar Man’ skeleton, the remains of at least six Late Palaeolithic human individuals (a child, two adolescents and three adults)^31^ have been recovered from the site, two of which have previously been directly radiocarbon dated. The skeletal remains have been shown to exhibit considerable humanly induced modification that can be attributed to cannibalistic practices and the production of ‘skull-cups’^31–33^. Previous analysis of radiocarbon dates from the human remains, as well as humanly modified faunal material, constrain the start of the Late Upper Palaeolithic occupation of the cave to 14,840–14,680 cal. bp (68.2% probability, IntCal09) and occupation lasted little more than 200 years^21,34^. We targeted one human temporal bone from Gough’s Cave (PV M 96544 (excavation numbers GC 86 (55) and GC 87 (60)) for ancient DNA (aDNA) analysis. The sample derives from the petrous part of the temporal bone (GC 87 (60)) and was recovered from a directly dated context along with other AMS dated human remains (Supplementary Information).

Kendrick’s Cave is located on Great Orme’s Head, a limestone massif in Llandudno, North Wales (Fig. 1). The site is known to have been used by people during the early part of the Late Glacial Interstadial (GI-1e) and has been associated with Magdalenian technologies due to the recovery of a proximal portion of a broken blade with *en éperon* butt preparation within the cave and a cut-marked bovine bone dated to ~14,500 cal. bp (OxA-17726, 12,310 ± 50 ^14^C bp)^22,35^. A decorated horse mandible dated to ~12,900 cal. bp (OxA-X-2185-26, 11,050 ± 90 ^14^C bp) shows that the site continued to be used later into the Late Glacial Interstadial. Beads made from the teeth of brown bear, aurochs and red deer, along with the remains of a minimum of four human individuals (three adults and one child) were also recovered from the site^36,37^. It has been argued that these incised and perforated artefacts share stylistic similarities with continental Late Palaeolithic art (Federmesser-Gruppen: the ‘penknife point’ culture)^38^. Five direct radiocarbon dates have been obtained from the human remains, providing dates from 11,990 to 11,905 ^14^C bp. However, only one of these dates included ultrafiltration in the pretreatment procedure^21^. Furthermore, the diet of these individuals has not previously been considered when calibrating these dates, which is necessary due to a marine and/or freshwater component in their diet^30^. Here, we redated four of the human bones and incorporated dietary information into the radiocarbon calibration (Results, Discussion and Supplementary Information). One human from Kendrick’s Cave (Kendricks_074) was also targeted for aDNA analysis. This sample derives from a mandibular first molar (M1) and the mandible from which this tooth derives has previously been AMS dated (with ultrafiltration) to 11,905 ± 50 ^14^C bp (OxA-17089).

The two sites, although chronologically close, show differences in funerary behaviour. Kendrick’s Cave has typically been interpreted as a burial site, in part due to the lack of faunal remains indicating food-processing activities or refuse at the site^30,36^. There is also no evidence of human modification on the Kendrick’s Cave human remains. In contrast, the human remains from Gough’s Cave were recovered from the same archaeological context as the faunal remains and both show significant human modification^32^. The human skeletal assemblage consists of scattered, highly fragmentary postcranial bones and relatively complete cranial vaults—some of which have, as already mentioned, been modified into skull-cups which has been interpreted as evidence for ritualistic cannibalism^31,33,39^.

## Results

### AMS dating and stable isotopic analysis

Four new ultrafiltered radiocarbon determinations were obtained from the Kendrick’s Cave human skeletal remains, which range from 11,990 ± 50 ^14^C BP (OxA-V-2794-27C) to 11,830 ± 50 ^14^C bp (OxA-V-2794-34C). These dates are all statistically indistinguishable from one another and from the previously published ultrafiltered date from the human mandible (OxA-17089, 11,905 ± 50 ^14^C bp) (error weighted mean = 11,932 ± 23 ^14^C bp, *T* = 6.8, d.f. = 4, *P* < 0.005). The dates fall within the range of the non-ultrafiltered previously published from the site (12,090 ± 90 ^14^C bp (OxA-6144) to 11,760 ± 90 ^14^C BP (OxA-7002)) but provide a more constrained date range, adding support to the interpretation that Kendrick’s Cave may have been used as a burial site. New δ^13^C and δ^15^N data obtained for these four samples were consistent with previous results attained. Previous isotopic analysis of the human remains suggested a diet which included marine and/or freshwater resources^30,40^, meaning the influence of reservoir effects on the AMS dates must be considered. A Bayesian mixing model was constructed to calculate the proportional influences of different food sources, using the programme FRUITS^41^, incorporating new and existing stable isotope data (Supplementary Information). A Bayesian model using OxCal (v.4.4) and the Mix_Curves function was applied to the Kendrick’s Cave humans and culturally modified faunal radiocarbon dates (Supplementary Information and Supplementary Table 7). This gives a boundary start date for human activity at the site of 16,410–14,070 cal. bp and a boundary end date of 13,730–13,140 cal. bp (95% confidence). When only the dates for the human remains are used in the model, however, the boundary start date is 14,100–13,460 cal. bp (95% confidence). The human individual used for aDNA analysis here (Kendricks_074) has a modelled date of 13,770–13,390 cal. bp (95% confidence, OxA-17089). However, as the stable isotope data from the Kendrick’s Cave humans has led to conflicting dietary interpretations regarding the proportions of marine and freshwater protein in the diet^30,40^, modelled dates should be treated with some caution until additional dietary information is available via, for example, compound specific isotope analysis or δ^34^S.

Additionally, a Bayesian modelling approach using OxCal (v.4.4) and the IntCal20 calibration curve was applied to the published radiocarbon dates of the Gough’s Cave humans and humanly modified fauna (Supplementary Information and Supplementary Table 6). The results of this give a boundary start date for the site of 15,070–14,850 cal. bp and a boundary end date of 14,960–14,610 cal. bp with a 95% confidence interval. This new calibration shifts the site occupation to being primarily before the rapid climate warming at the start of the Late Glacial Interstadial (~14,700 bp) as recorded in the Greenland ice cores (GI-1e)^42^, although it is possible that the end of the occupation may have occurred just after the onset of the Late Glacial Interstadial.

The new AMS dates and recalibration of dates for both the Gough’s Cave site and Kendrick’s Cave individual demonstrate that although the sites are close in age, there is no overlap between the dates of the Gough’s Cave occupation and the Kendrick’s Cave humans (at 95% confidence). However, there is an overlap between the Kendrick’s Cave humanly modified bovid bone and the Gough’s Cave human occupation (at 95.4% confidence), indicating that the two sites were occupied contemporaneously. When comparing the boundary end date for the Gough’s Cave occupation to the date of the Kendrick specimen sampled for aDNA, there is at least 600 years age difference when 100% terrestrial-based diet is assumed for both. However, this is probably an underestimate as incorporating the reservoir effect for marine and/or freshwater dietary components for the Kendrick’s individual would make it younger in date and increase the age gap between the two individuals to a minimum of 840 years and potentially up to ~1,200 years (Supplementary Information).

### aDNA analyses

We recovered 15,497 and 9,702 unique mitochondrial DNA fragments, respectively, for the Gough’s Cave and Kendrick’s Cave individuals, resulting in 53.8-fold and 34.8-fold average coverage of their mtDNA genomes. We also recovered 30,587,614 and 29,326,159 nuclear DNA fragments from Gough’s Cave and Kendrick’s Cave individuals by direct shotgun sequencing, amounting to an average of 0.53-fold and 0.48-fold genomic coverage for the two individuals, respectively. The proportion of DNA fragments mapping to the human reference genome for the Gough’s Cave and Kendrick’s Cave individuals was 23% and 18%, with an average fragment length of 62 base pairs (bp) and 63 bp, respectively. Each DNA fragment from the two individuals was seen on average between 1.95 and 2.29 times in sequencing, translating to clonality of 49% and 56%.

We reconstructed complete mtDNA sequences of both individuals and determined their haplogroups using HaploGrep^43^ and the Phylotree database (build 17). The Gough’s Cave individual carries substitutions that define the haplogroup U8a (0.97 posterior support) and the Kendrick’s Cave individual carries substitutions that define the haplogroup U5a2 (1.0 posterior support). The U8a haplogroup has not previously been detected in British early prehistoric individuals but has been identified in Magdalenian individuals elsewhere in Europe, for example at Hohle Fels and Brillenhöhle, Germany, and the Goyet Q2 individual from Belgium^8^. A number of British Mesolithic individuals have previously been found to carry the U5 mt haplogroup, including one from Kent’s Cavern which has also been determined as U5a2 (ref. ^25^).

We determined the sex of the two individuals by assessing the number of DNA fragments that align to the X and Y chromosomes^44^. We found that the individual from Gough’s Cave is female and the individual from Kendrick’s Cave is male (Supplementary Information). The Kendrick’s Cave skeletal material was referred to as representing ‘men’^36^ in original descriptions of the site but no formal osteological assessment of biological sex was undertaken.

**Fig. 2:**
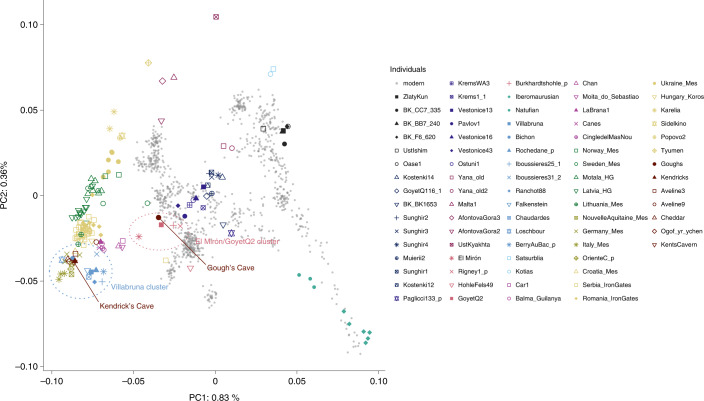
Principal component analysis (PCA) of ancient individuals. PCA of 1,087 present-day West Eurasians genotyped on 597,573 SNPs (grey points) with 168 ancient hunter gatherers (Supplementary Table 3) from West Eurasia and North Africa older than ~7,000 cal. bp and with more than 30,000 SNPs projected onto the plane. PC1, principal component one; PC2, principal component two.

**Fig. 3:**
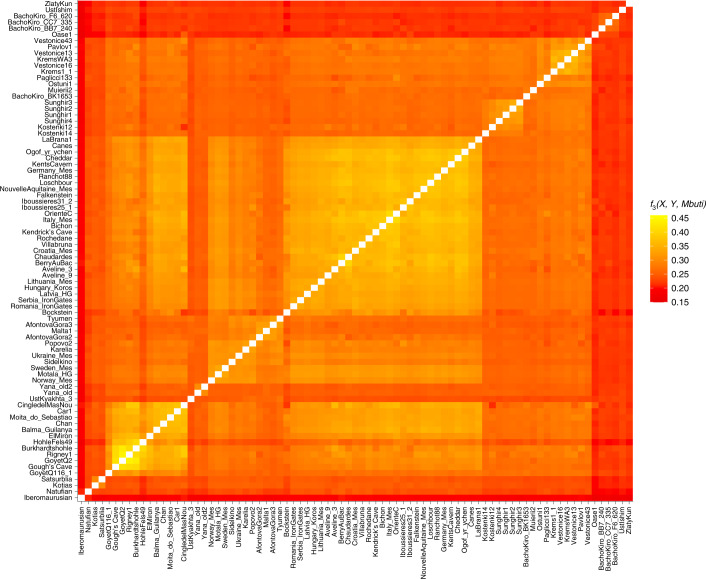
Genetic clustering of ancient individuals, including Gough’s Cave and Kendrick’s Cave individuals, calculated as *f*_3_(ancient1, ancient2; Mbuti). Lighter colours correspond to higher *f*_3_ values and indicate a higher shared genetic drift between pairs of ancient individuals/populations. The *X*-axis corresponds to ancient1 and the *Y*-axis to ancient2 individuals for the calculation *f*_3_(ancient 1, ancient 2; Mbuti). The order of individuals is based on the hierarchical clustering of *f*_3_ values, and ancient individuals included in this analysis are the same as those in Figure 2. Plotted *f*_3_ values were calculated using ADMIXTOOLS as implemented in admixr.

The data generated for the Gough’s and Kendrick’s Cave individuals contains 506,151 and 476,347 single nucleotide polymorphisms (SNPs) respectively, overlapping the ‘1240k’ SNP panel^45,46^ informative of the genetic relationships amongst ancient and present-day humans. In a principal component analysis^47,48^ (Supplementary Information), the Gough’s Cave individual falls close to the ~15,000-year-old Goyet Q2 individual from Belgium, whereas the Kendrick’s individual clusters with individuals with predominantly WHG-like ancestry, including British Mesolithic individuals (Fig. 2). By computing *f*_3_ statistics in the form of *f*_3_(Gough’s/Kendrick’s, ancient; Mbuti), which measures the amount of shared genetic drift between pairs of ancient individuals after their separation from an outgroup (in this case Mbuti from ref. ^49^), we again found that the Gough’s Cave individual shares most drift with the individuals belonging to the ~19,000–14,000-year-old Goyet Q2 genetic cluster, whereas the Kendrick’s Cave individual shares most drift with the individuals belonging to the ~14,000–7,000-year-old Villabruna genetic cluster (Fig. 3).

We further confirmed these affinities directly with *D* statistics by calculating *D*(ancient_1_, ancient_2_; Gough’s/Kendrick’s, Mbuti). Again, we found that the Gough’s Cave individual shares significantly more alleles with the members of the Goyet Q2 genetic cluster than the Villabruna genetic cluster. In contrast, the Kendrick’s Cave individual, as well as Mesolithic individuals from Britain^25^, share significantly more alleles with the members of the Villabruna cluster than with the members of the Goyet Q2 cluster.

**Fig. 4:**
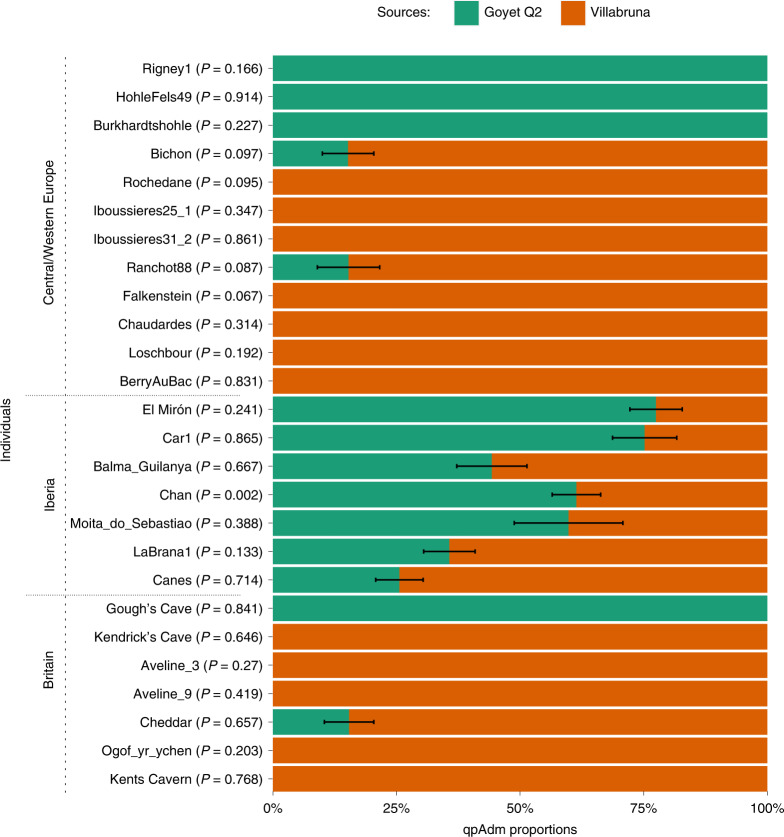
Modelling West Eurasian hunter-gatherer individuals (*n* = 26) as having a single-source or a two-source ancestry with qpAdm, using the lowest rank model. The ~15,140-year-old Goyet Q2 individual from Belgium and the ~14,060-year-old Villabruna individual from Italy were used as potential sources. Within each geographical area (Central/Western Europe, Iberia and Britain) individuals are ordered on the basis of their chronological age from oldest to youngest. Standard errors (s.e.) were calculated using weighted block jackknife and a block size of 5 Mb. Here, s.e. indicates ±1 s.e.

We used admixture modelling with qpWave and qpAdm^50,51^ to explore the ancestry of the Gough’s and Kendrick’s Cave individuals in more detail. We used the Goyet Q2 and Villabruna individuals as potential source populations. We modelled the Gough’s individual as having single-source Goyet Q2 ancestry (*P* = 0.841) and the Kendrick’s Cave individual as having single-source Villabruna ancestry (*P* = 0.646). All other single-source models can be rejected (*P* << 0.001). Interestingly, all Mesolithic individuals from Britain, except Cheddar Man^25^, can also be modelled as having un-admixed Villabruna ancestry in this analysis. Cheddar Man, an individual also recovered from Gough’s Cave and dating to 10,564–9,915 cal. bp (IntCal20, 9,100 ± 100 ^14^C bp (OxA-814)), is instead best modelled as having 84.6% (±0.5%) Villabruna-related ancestry and 15.4% (±0.5%) Goyet Q2-related ancestry (Fig. 4).

However, it should be noted that although single-source models can explain the data from Mesolithic individuals from Britain^25^ and no additional model complexity is needed, two-source models of genetic ancestry of these previously published Mesolithic individuals cannot be fully rejected either. In these two-source models, Villabruna ancestry remains the predominant component for all individuals, corresponding to between 74.8% (±9.7%, Aveline9) and 93.2% (±6.7%, Ogof yr ychen) of the genetic ancestry of these individuals. In contrast, the Gough’s Cave Magdalenian individual can only be modelled as having a single-source Goyet Q2 ancestry and the Kendrick’s Cave individual can be modelled as having a single-source Villabruna ancestry, with two-source models either strongly rejected or with estimated admixture proportions outside the range 0–100%, indicative of poor fit (Supplementary Fig. 12).

## Discussion

Combined, the genetic results and AMS dates from these individuals indicate the presence of two genetically distinct groups in Britain in the Late Glacial period. This is evident through both the differential mitochondrial haplogroups of the two individuals analysed here and also through their distinctive ancestral patterns. The Gough’s Cave individual shows clear affinity to Goyet Q2 ancestry, whereas the Kendrick’s Cave individual shows affinity to Villabruna (WHG). It is also interesting to note the lack of genetic admixture at Gough’s Cave given the lithics assemblage is of mixed origin, containing both late Magdalenian and early Federmesser-Gruppen technologies. Furthermore, the single culturally identifiable lithic from Kendrick’s Cave has been ascribed to the Magdalenian, whereas Villabruna ancestry has previously been associated with Epigravettian and Azilian/Federmesser cultures. However, the incised and perforated artefacts at Kendrick’s Cave do bear stylistic similarities with continental art linked to the Federmesser-Gruppen culture^38^. It may be that boundaries between cultural and genetic affinities break down at this time or alternatively culturally and genetically distinct groups are present at both Gough’s Cave and Kendrick’s Cave but the evidence is at a resolution we at present cannot chronologically resolve. However, our analyses demonstrate that Villabruna ancestry was already present within Britain during the Late Glacial. This suggests that the emergence of Villabruna ancestry in Britain predates the Holocene. It is possible that there may have been more than one migration of Villabruna ancestry into Britain however—perhaps, for example, a secondary migration at the start of the Mesolithic period—but our data do not currently have the resolution to comment on this possibility.

It is important to note, however, that the temperate climate of the Late Glacial Interstadial and the early Holocene was punctuated by the Younger Dryas Stadial (~12,900–11,700 bp, broadly equivalent to GS-1), when temperatures were notably colder, ice sheets expanded in Scotland and reindeer once again became the dominant fauna in the cave sites of southwest Britain. Currently, there are no radiocarbon determinations documenting human presence in the British Isles during the Younger Dryas^52^. Although this may be the result of taphonomy and preservation issues, if a gap in human presence is real then this indicates more than one migration of Villabruna ancestry into Britain may have occurred. On the basis of the data currently available, whilst the Late Palaeolithic Kendrick’s Cave individual indicates it has single-source Villabruna ancestry, some British Mesolithic individuals show significantly more affinity to Cheddar Man, which can be modelled as having two-source Goyet Q2 and Villabruna ancestry (Supplementary Information). This, therefore, suggests that a degree of genetic change may have occurred in tandem with the substantial cultural change seen with the emergence of the Mesolithic in Britain, to the limits of our current resolution.

Interestingly, these two genetically distinct Late Upper Palaeolithic populations, present in Britain ~600 and 1,200 years apart, appear to have also had isotopically different diets. The individuals from Kendrick’s Cave show evidence of intensive consumption of marine and freshwater foods, including high trophic level marine mammals^30,40,53^. In contrast, the Gough’s Cave human skeletal assemblage shows no evidence for marine or freshwater resource consumption and instead diet was based primarily on terrestrial herbivores, specifically red deer and bovids but also horses^28,54^. However, this assumes that the cannibalized individuals were also those consuming the faunal remains recovered from the site. In tandem with this, it is interesting to note that whilst there is evidence of cannibalism and secondary treatment of human material at Gough’s Cave (also found at other Magdalenian sites such as Brillenhöhle and Höhle Fels in Germany and Mazsycka Cave in Poland^55,56^), Kendrick’s Cave has been interpreted as being used as a possible burial site, associated with important portable art items such as the decorated horse mandible^57^. Combined, these lines of evidence support the interpretation that at least two different human groups, with different genetic affinities and dietary and cultural behaviours, were present in Britain during the Late Glacial.

Determining potential sources for these populations is, however, complex. During this period, Britain was connected via Doggerland to the main European continent. Despite this, the Late Glacial Channel River was probably difficult to cross at its more southwesterly points, such as from the Paris Basin and is suggested to have created (seasonal) barriers to movement^58,59^. Instead, it has been proposed that populations arriving in Britain during the Late Glacial may have taken a more easterly route, between the Channel River and the Palaeo-Elbe catchment, possibly across an area of higher ground linking Britain with Belgium and the Netherlands^59–61^. These hypotheses are difficult to test, however, due to the lack of Late Palaeolithic remains suitable for aDNA and AMS dating preserved in these regions.

Nonetheless, our qpAdm modelling indicates that Goyet Q2 ancestry persisted in Britain until at least 15,070 cal. bp and potentially as late as 14,610 cal. bp based on the modelled boundary start and end dates for Gough’s Cave. The appearance of people in southwest England before the Interstadial warming and soon after the earliest evidence for reindeer and horse returned to the landscape, combined with this Goyet Q2 ancestry, suggests that these people may have come from Magdalenian populations that had remained isolated during the LGM and early Late Glacial from more southerly populations where admixed Goyet Q2 and Villabruna ancestry is evident^8^. Indeed, it is perhaps the post-LGM climate amelioration and Late Glacial rapid climatic warming, causing key cold-adapted prey species to contract to more northerly latitudes, which facilitated this—in effect, a retreat to the north by Magdalenian cold-adapted populations. From at least 13,800 to 13,240 cal. bp (Kendricks_074, 95% confidence, OxA-17089) however, Villabruna (WHG) ancestry appears in Britain and persists into the Mesolithic, being replaced only at the start of the Neolithic with the emergence of agriculture. The source population for this ancestry and its route into Britain remains unclear but the rapid climatic warming of the Late Glacial Interstadial, which resulted in substantial environmental change, may have provided new ecological opportunities for human populations. Similarly, these environmental developments may have placed considerable pressures on cold-adapted fauna and on the people who specialized in their exploitation.

## Conclusions

We extend the scope of European palaeogenomics here by sequencing the first Palaeolithic human skeletal material from Britain. Furthermore, the genetic data generated within this study clearly demonstrate that there appears to have been dual genetic ancestries present in Britain during the Late Glacial period. New AMS dating and recalibration of existing dates generated on material from the two sites studied here also indicate that these two genetically distinct populations in Late Upper Palaeolithic Britain were close in date, potentially ~600 years apart. Interestingly, dual Late Pleistocene genetic ancestry has also been demonstrated in Iberian hunter gatherers^9^. However, although admixed Goyet Q2 and Villabruna ancestry can be seen in southern Europe at El Mirón from at least ~18,770 cal. bp (ref. ^8^; Fig. 1), this signature of admixture is not visible in British individuals, thereby suggesting a more significant genetic turnover or replacement in northwestern Europe than in the southwest.

In addition, we demonstrate that the Gough’s Cave and Kendrick’s Cave individuals, despite being close in date, differ not only in their genetic ancestry profiles but also in their mortuary practices and their diets, as evidenced through stable isotopic analyses. This presents a picture of a dynamic and varied Late Glacial period within Britain, with changes occurring in the Late Upper Palaeolithic in diet, funerary behaviours, technologies and genetic affinity at a time of rapid environmental and ecological change. With the addition of our data to the existing knowledge of early prehistoric genetics in Britain^24,25^, the emerging scenario is one of multiple genetic population turnover events in the United Kingdom. This can be seen to reflect a dynamic, changing population throughout British early prehistory and which mirrors the events seen across continental Europe.

The lack of human remains from Late Pleistocene Britain, combined with DNA preservational limits, means analyses of the period will always be limited. We demonstrate here, however, that it is possible to obtain useful genetic information from Late Glacial human skeletal material in Britain and that these data can further our understandings of early occupation of the British Isles, population movement, interactions with the continental Europe and potential population replacements.

## Methods

AMS dating of the Kendrick’s Cave material was undertaken at the Oxford Radiocarbon Accelerator Unit (ORAU) following sample preparation at University College London (UCL) using a modified version of the ORAU collagen extraction procedure^62^. Stable carbon (δ^13^C) and nitrogen (δ^15^N) isotope analysis was also conducted on the collagen to confirm previous isotope results^30^ (Supplementary Information).

Ancient DNA extraction and library preparation was undertaken in the dedicated aDNA laboratory at the Natural History Museum, London. Between 0.027 and 0.030 g of finely drilled bone or cementum powder was used from each specimen. DNA was extracted using a modified version of the protocol outlined by ref. ^63^, designed specifically for short DNA fragments. Library preparation followed a modified version of the Meyer and Kircher^64^ protocol, including the partial uracil–DNA–glycosylase treatment described in ref. ^65^. All libraries were double indexed^66^ and sequenced on a HiSeq 4000 platform (100 bp paired-end) at the Francis Crick Institute, London (Supplementary Information).

Stringent methods were taken during laboratory work to minimize DNA contamination and negative controls were processed in parallel with samples (Supplementary Information). We strictly assessed the authenticity of the data generated. Sequenced DNA fragments from both individuals show elevated frequencies of substitution patterns at the ends of the alignments characteristic of aDNA^67^, assessed through PMDtools^68^ (Supplementary Information).

To directly compare Gough’s and Kendrick’s Cave individuals to a broader set of ancient modern humans from the same time period, we intersected the generated shotgun data with ~1.23 million (‘1240k’) SNPs informative of the genetic relationships among ancient and present-day humans^45,46^ using BEDtools (v.2.23.0)^69^. We used bam-caller (https://github.com/bodkan/bam-caller, v.0.1) to generate ‘pseudo-haploid’ calls of both individuals by picking a base at each position that was covered by at least one DNA fragment of at least 35 bp with a mapping quality of at least 25 (MQ ≥ 25). We merged these data with previously reported data of ancient and present-day humans compiled in the Allen Ancient DNA resource (release 20 January 2021, v.44.3, https://reich.hms.harvard.edu/allen-ancient-dna-resource-aadr-downloadable-genotypes-present-day-and-ancient-dna-data) (Supplementary Table 3).

We computed principal components of 1,087 present-day West Eurasians genotyped on 597,573 SNPs of the Affymetrix Human Origins array^50^ using smartpca from the EIGENSOFT package and projected 168 ancient hunter gatherers from West Eurasia and North Africa older than ~7,000 cal. bp (Supplementary Table 3) on the first two components using the ‘lsqproject’ option. The *f* statistics and *D* statistics were computed with ADMIXTOOLS (v.5.1)^50^ as implemented in the R-package admixr^70^ and standard errors were calculated using a weighted block jackknife^50,71^ with equally sized blocks of 5 Mb.

We used qpAdm (v.650) from ADMIXTOOLS^50^ to further model the ancestry of the Gough’s Cave and Kendrick’s Cave individuals, as well as other individuals from the same time period in western Eurasia. We explored one- and two-source models by using the ~15,140-year-old Goyet Q2 individual from Belgium 8,54 and the ~14,060-year-old Villabruna individual from Italy^8^ as potential source populations and Ust’Ishim^72^, Goyet Q116-1^8^, Mal’ta 1^73^, Natufian^74^, Koros^75^, Mota^76^, Onge, Han, Papuan, Karitiana and Mbuti individuals^49^ as reference populations^77^.

### Reporting summary

Further information on research design is available in the Nature Research Reporting Summary linked to this article.

## Supplementary information

Supplementary Information Supplementary Methods, Figs. 1–12 and Tables 1 and 2.

Reporting Summary

Supplementary Tables Supplementary Tables 3–8.

## Data Availability

BAM files (one file per library) have been deposited in the European Nucleotide Archive under study accession number PRJEB52727.
