# Supplementary material for: Dual ancestries and ecologies of the Late Glacial Palaeolithic in Britain
Source: Nat Ecol Evol. 2022 Oct 24;6(11):1658–68. doi: 10.1038/s41559-022-01883-z (PMC9630104; doi:10.1038/s41559-022-01883-z)
Supplement: Supplementary file 1 — Supplementary Methods, Figs. 1–12 and Tables 1 and 2. [file 41559_2022_1883_MOESM1_ESM.pdf]

nature ecology & evolution

Article https://doi.org/10.1038/s41559-022-01883-z

Dual ancestries and ecologies of the Late Glacial Palaeolithic in Britain

In the format provided by the authors and unedited

Supplementary Information

Site background and sample information

Gough's Cave

Gough's Cave is part of a large cave system situated at the mouth of Cheddar Gorge in the Mendip Hills, Somerset, southwest England. The site was explored archaeologically as early as 1903, with the discovery of the Early Mesolithic 'Cheddar Man' burial, but was most extensively excavated 1927–1931 by R.F. Parry, the land agent for the Marquess of Bath (Stringer 2000; Jacobi and Higham 2009). Gough's Cave is believed to have both Final Magdalenian (known locally as Creswellian) and Federmesser-Gruppen occupations, and the start of occupation at the site coincides with the beginning of the Late Glacial Interstadial (c. 14,700 cal. BP, broadly synchronous with the Greenland Interstadial (GI) 1e), a period of significant climatic warming (Jacobi and Higham 2009; Jacobi and Higham 2011) (see also 'AMS dating' section below). The site is particularly famous due to its lithic and faunal assemblages being the largest of any British Palaeolithic cave site (Jacobi and Higham 2009; Jacobi and Higham 2011), and has been variously suggested to be a base-camp, an aggregation site, and a site for the hunting of horse and red deer (Currant 1986; Jacobi and Higham 2009). Bone, antler and ivory also served as raw materials for artefact manufacture, such as perforated *bâtons*, mammoth ivory javelin heads, 'blanks' of swan and hare bone from which needles were cut, awls, a needle, fox tooth beads, sea shells, incised ivory, amber and scratched pebbles (Charles 1989; Lucas et al. 2019).

The Palaeolithic artefacts and faunal remains from Gough's Cave were recovered from the 'Cave-Earth and Breccia' layer by Parry, which is believed to be up to 4.3m in thickness (Parry 1929; Jacobi and Higham 2011). Fauna present at the site include species such as wolf (*Canis lupus*), collared lemming (*Dicrostonyx torquatus*), horse (*Equus ferus*), red deer (*Cervus elaphus*), reindeer (*Rangifer tarandus*), aurochs (cf. *Bos primigenius*) and brown bear (*Ursus arctos*), amongst others – and much of the faunal assemblage shows evidence of butchery and deliberate breakage (Currant 1986; Currant and Jacobi 2001). Alongside this, a number of artefacts manufactured from faunal remains were also recovered, including three *bâtons percés* made from reindeer antler, awls made from the tibiae of mountain hares (*Lepus timidus*), and a bevel-based rod made from mammoth (*Mammuthus primigenius*) ivory (Jacobi and Higham 2009; Jacobi and Higham 2011). The lithic assemblage from the site contains over 2200 artefacts and is dominated by blades, including bi-truncated trapezoidal backed blades and a significant number with 'en éperon' butt preparation, as well as tools such as burins, end-scrapers, piercers, and curve-backed points (Jacobi 2004).

The skeletal remains of at least six Late Palaeolithic human individuals (a child, two adolescents and three adults) have also been recovered from the site. In this study, DNA was recovered

from the petrous bone of one of these individuals (Supplementary Table 1). No new isotope or radiocarbon dates were obtained from Gough's Cave for this study. The published radiocarbon dates and isotope data used here in date and dietary reconstruction are provided in Supplementary Table 4.

Kendrick's Cave

Kendrick's Cave is located on Great Orme's Head, a limestone massif in Llandudno, North Wales. The site was originally discovered in the 19th century by a lapidary, Thomas Kendrick, whilst enlarging his workshop located within the small cavern (Eskrigge 1879). It is important to note, however, that there are two caves - an upper and a lower - both of which have generated archaeological assemblages. Unfortunately, the literature is often unclear as to which material was recovered from which cave. An original report about the work of Thomas Kendrick, after whom the site is named, suggests that it is the lower of the two caves from which the human remains and artefacts were recovered (Eskrigge 1879). The report describes the human remains as burials coming from the cave earth with a 'chimney-like' feature extending upwards in the cave (Eskrigge 1879). Later investigations of the site have also suggested that the majority of the Palaeolithic material originated from the lower cave (Rees and Nash 2017). However, the Upper Kendrick's Cave was explored by Melvyn Davies 1977-1979, and he instead attributed the Palaeolithic finds to the Upper Cave (Davies 1989).

The site is known to have been utilised in GI-1e by people using Magdalenian technologies, due to the recovery of a proximal portion of a broken blade with '*en éperon*' butt preparation within the cave, and also subsequent AMS dating of a cut-marked bovine bone (Davies 1989; R. Jacobi and Higham 2011). However, Kendrick's Cave has generally been considered as a burial site, rather than an occupation site, supported by the small number of faunal remains recovered from the cave (Dawkins 1880; Richards et al. 2005). However, the lack of clarity concerning their context make their interpretation as a deliberate burial somewhat speculative. Similarly to Gough's Cave, the lack of precise documentation makes distinction of different occupation events based on vertical and/or horizontal stratigraphic positions difficult. However, based on techno-typological and ecological considerations, radiocarbon dating, and stratigraphic observations, more than one visit to the site seems plausible.

The recovery of the Upper Palaeolithic artefacts at the site, as well as some faunal remains, are believed to all derive from a limestone breccia excavated by Kendrick (Sieveking 1971). A number of artefacts manufactured from faunal remains were recovered from this context, including incised and perforated bovid, deer and bear teeth, and most famously, a decorated horse (*Equus ferus*) mandible (Dawkins 1880; Sieveking 1971). Some of these artefacts date to the second part of the Late Glacial interstadial (e.g. an incised roe deer metacarpal dated to 13,790-13,510 cal. BP (OxA-6116, 11,795 ±65 14C BP), and the decorated horse mandible, dated to 13,105 - 12,765 cal. BP (OxA-X-2185-26, 11,050 ±90 14C BP) (Richards et al. 2005) (http://www.britishmuseum.org/research/collection_online/collection_object_details.aspx?objectId=1599124&partId=1). However, a perforated canine of badger has been dated to the

Pleistocene - Holocene transition, 11,730-11,220 cal. BP (OxA-5862, 9945 ±75 14C BP, Bronk-Ramsey et al 2002). It has been argued that these incised and perforated artefacts bear stylistic similarities with continental Late Palaeolithic art (Federmessergruppen: the 'penknife point' culture) (Pettitt 2003).

Also recovered from the limestone breccia were the partial remains of at least four human skeletons - three adults and one child (Dawkins 1880). Stable isotope analysis and radiocarbon dating was previously undertaken on five elements from these specimens (Richards et al. 2005; R. M. Jacobi and Higham 2009) (Supplementary Tables 4 and 5). It is likely two of the five dates come from different elements of a single individual adult. For four of the previous radiocarbon dates, the sample pre-treatment protocol used did not include an ultrafiltration step, which is known to remove low molecular weight contaminants and therefore impact radiocarbon age determination (Higham, Jacobi, and Bronk Ramsey 2006). Due to this, we re-sampled these four skeletal elements for AMS dating and stable isotope analysis in order to refine the timing of the human occupation of Kendrick's Cave (Supplementary Table 5). DNA was recovered from a tooth root extracted from the mandible of one of these adult individuals (074) (Supplementary Table 1).

Museum Number	DNA Lab Code	Project Code	AMS Lab Code	Site	Element	Material	Weight used /g
PV M 96544 (excavation number: GC 87 (60))	SB392	N/A	Not directly dated	Gough's Cave	Petrous	Bone	0.027
074	SC112/ SC206	UPN-637	OxA-17089	Kendrick's Cave	Tooth (Mandibular M1)	Cementum	0.030

Supplementary Table 1: Details of samples utilised for aDNA analysis in this study

Collagen extraction for isotope analysis and radiocarbon dating

A small sample of bone was collected from each specimen using a dental drill with a tungsten burr attachment. Samples were prepared at University College London (UCL) using a modified version of the Oxford Radiocarbon Accelerator Unit (ORAU) collagen extraction procedure (Brock et al. 2010), which is based on a modified version of the (Longin 1971) protocol. All samples were treated with 0.5M hydrochloric acid (HCl) at 4°C until fully demineralised and then thoroughly rinsed with ultrapure water. Samples were then treated with 0.1M sodium hydroxide (30mins) and 0.5M HCl (1hr) to remove humic contaminants (Szpak, Metcalfe, and Macdonald 2017), again being thoroughly rinsed with ultrapure water between reagents. All samples were then gelatinised in pH3 HCl solution at 75°C for 48hrs and filtered using a pre-cleaned Ezee-

filter. The filtrate was then passed through a pre-cleaned 15–30 kD ultrafilter, with the >30 kD fraction collected and freeze-dried (AF method).

Isotopic analysis of collagen

Between 1.2–1.5mg aliquots of freeze-dried collagen were weighed into tin capsules and analysed using a Delta V Advantage continuous-flow isotope ratio mass spectrometer coupled via a ConfloIV to an EA IsoLink elemental analyser (Thermo Fisher Scientific, Bremen) at the Scottish Universities Environmental Research Centre (SUERC). For every ten unknown samples, three in-house standards that are calibrated to the International Atomic Energy Agency (IAEA) reference materials USGS40 (L-glutamic acid, $\delta^{13}C_{VPDB}$ = –26.4‰, $\delta^{15}N_{AIR}$ = –4.5‰), USGS41 (L-glutamic acid, $\delta^{13}C_{VPDB}$ = +37.6‰, $\delta^{15}N_{AIR}$ = +47.6‰), USGS43 (Indian Human Hair: $\delta^{15}N_{AIR}$ = +8.44‰, $\delta^{13}C_{VPDB}$ = –21.28‰) were analysed (Sayle et al. 2019). Results are reported as per mil (‰) relative to the internationally accepted standards VPDB and AIR. Measurement uncertainty was determined to be ±0.1‰ for $\delta^{13}C$ and ±0.2‰ for $\delta^{15}N$ on the basis of repeated measurements of an in-house bone collagen standard and a certified fish gelatin standard (Elemental Microanalysis, UK). Each sample was analysed in duplicate with the exception of one sample (UPN-643, Museum No 069) and reproducibility was better than ±0.1‰ for $\delta^{13}C$ and ±0.2‰ for $\delta^{15}N$. Results for this isotopic work are given in Supplementary Table 5. The new $\delta^{13}C$ and $\delta^{15}N$ values obtained here were consistent with previous data obtained from these specimens.

Dietary reconstruction

We considered the diets of both the Gough's (n=4) and Kendrick's (n=5) human samples using published data and additional stable isotope analysis ($\delta^{13}C$ and $\delta^{15}N$) (Supplementary Tables 4 and 5). Previous dietary studies at Gough's Cave have indicated no evidence of marine of freshwater resource consumption, and instead the diet of individuals at the site has been shown to be based primarily on terrestrial herbivores, most likely red deer and bovids (Richards et al. 2000; Stevens, Jacobi, and Higham 2010/1). In contrast, the individuals at Kendrick's Cave's have previously been shown to have a mixed terrestrial and aquatic diet. Initial investigations suggested a diet focused on marine and terrestrial mammals (Richards et al. 2005), however later studies argued the diet also included a freshwater fish component (Bocherens and Drucker 2006; Pickard and Bonsall 2020). During the Late Glacial period, with sea levels 15 to 20m lower than today along the North Wales coast (Shennan et al. 2006), estuarine environments probably existed close to Kendrick's Cave. This likely allowed humans who were utilising the site to exploit resources from both marine and freshwater environments, along with terrestrial food sources (Pickard and Bonsall 2020).

Due to the mixed nature of the Kendrick's Cave individuals' diets, a Bayesian mixing model was constructed to calculate the proportional influences of different food sources, using the programme FRUITS (Fernandes et al. 2014), and incorporating new stable isotope data from

this study (Supplementary Table 5). Although such an approach has previously been undertaken (Pickard and Bonsall 2020), we use a more chronologically restricted isotope dataset (Supplementary Table 6) to establish the potential terrestrial dietary input, as terrestrial faunal nitrogen isotope values are known to change in response to climatic/environmental change during the Late Glacial (Stevens and Hedges 2004). A simple routed model with six food groups as outlined in (Pickard and Bonsall 2020) was generated, and then incorporated into the three main overall food groups of 'freshwater', 'marine' and 'terrestrial' using values published in (Pickard and Bonsall 2020) (Supplementary Figure 1). FRUITS parameter specifications used in the model were: diet-collagen offsets 4.8 ± 0.5% for $\delta^{13}C$ and 5.5 ± 0.5% for $\delta^{15}N$ (Fernandes et al. 2014) dietary routing: 74 ± 4% for $\delta^{13}C$ and 100% for $\delta^{15}N$ from dietary protein, 26% for $\delta^{13}C$ from energy macronutrients; food isotope values were taken from (Pickard and Bonsall 2020) with refined terrestrial meat isotope values presented in Supplementary Table 6. Prior information entered into FRUITS followed (Fernandes et al. 2014). The total % freshwater, marine and terrestrial dietary inputs were calculated by combining the $\delta^{13}C$ values of the six food groups into the appropriate overarching group by summing the mean, with the uncertainty calculated using the square root of the sum of squares (Supplementary Figure 1 and Supplementary Table 5). This process was repeated by combining the $\delta^{15}N$ values (Supplementary Table 5). The calculated % freshwater, marine and terrestrial dietary inputs based on the $\delta^{13}C$ results were used when calibrating the radiocarbon dates (see AMS dating section).

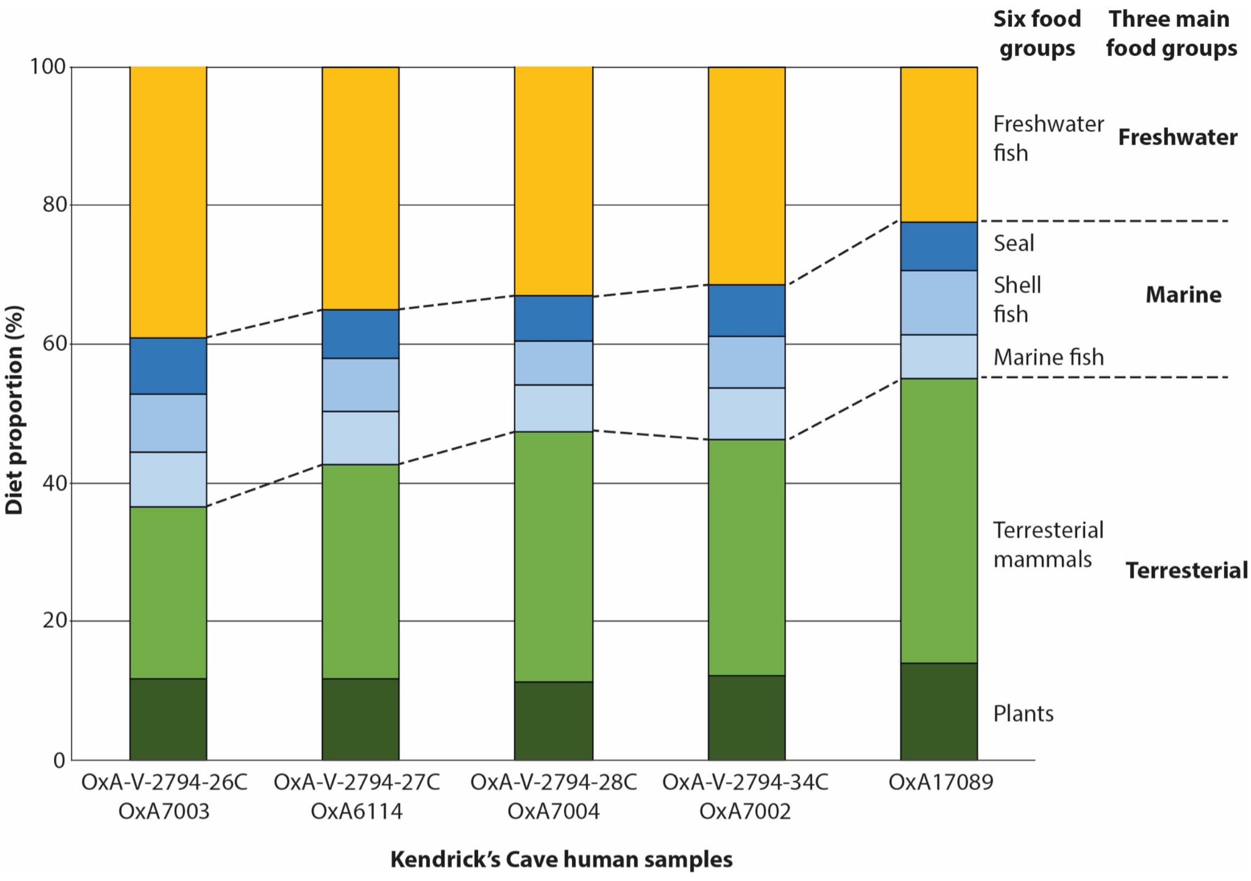

Supplementary Figure 1: Results of proposed food intake for Kendrick's Cave human individuals using a simple routed FRUITS model based on the human $\delta^{13}C$ results and the $\delta^{13}C$ of potential food sources (Supplementary Tables 5 and 6)

Supplementary Figure 1 and Supplementary Table 5 present the results of the proportion of dietary sources for individuals from Kendrick's Cave. The results show a mixed diet varying between terrestrial, marine and freshwater sources, as demonstrated in (Pickard and Bonsall 2020). The terrestrial food source equates to the greatest contribution of the Kendrick's Cave human diet with a plant food contribution 11 ± 10% to 14 ± 12%, and terrestrial mammal contribution from 25 ± 13% to 41 ± 15%. However, marine and freshwater resources also still appear to be important, with marine contributions ranging from 20 ± 10% to 25 ± 11%, and freshwater dietary contributions of 23 ± 14% to 39 ± 16%.

AMS dating

Both the Gough's Cave and Kendrick's Cave assemblages have a number of radiocarbon dates associated with them, including some recent ultrafiltered AMS determinations. Bayesian modelling of ultrafiltered dates from human remains and culturally modified faunal remains from Gough's Cave was previously undertaken by (Jacobi and Higham 2011), and suggested human use of the site began at 14,950–14,750 cal. BP (IntCal09). However, this was prior to the publication of the new IntCal20 calibration curve (Reimer et al. 2020). AMS dates undertaken on bovid bones from Kendrick's Cave (of which one specimen was cut-marked), have placed Later Upper Palaeolithic activity at the site at 14,500–14,040 cal. BP (IntCal09, unmodelled 65% confidence interval) (Jacobi and Higham 2011).

The Gough's Cave individual sampled here for aDNA analysis (PV M 96544 (excavation numbers GC 86 (55) and GC 87 (60)) has not been directly dated, but was recovered from the same context as human skeletal remains that have been dated to 15,175 - 14325 cal. BP (OxA-17848, 12,485 ±50 14C BP, OxA-17849, 12,590 ±50 14C BP). The Kendrick's Cave human individual analysed here genetically has been directly AMS dated. To refine the timing of the human occupation of Kendrick's Cave four skeletal elements previously dated were also re-sampled for AMS dating here. Dates on samples prepared in the laboratory at UCL were further corrected for laboratory background carbon as outlined in (Reade et al. 2020). Corrected dates are indicated by a "C". Due to the publication of the new IntCal20 calibration curve (Reimer et al. 2020) and the isotopic data available from both sites, the dates for these individuals and all the dated Late Glacial Gough's Cave and Kendrick's Cave individuals were recalibrated with IntCal20. Several steps were undertaken to provide accurate calibrated ages, particularly at Kendrick's Cave. All calibrated ages presented here are reported using 2 sigma or 95.4% confidence range (Supplementary Table 4).

Appropriate reservoir effect values

The FRUITS results generated for the Kendrick's Cave individuals have chronological implications for the ages of human remains at the site, particularly with the influence of marine and freshwater food sources. The influence of both a freshwater reservoir effect (FRE) and a marine reservoir effect (MRE) can be large (e.g. FRE 400-500 ^{14}C years; (Meadows et al. 2020)). Consequently, appropriate reservoir values need to be defined for both FRE and MRE. There are currently no local FRE values for the Late Pleistocene in the British Isles due to the scarcity of freshwater fish bones at archaeological sites. In addition, there are no modern FRE values for the Menai Strait (which is believed to be freshwater by (Pickard and Bonsall 2020) during the occupation of Kendrick's Cave) due to inundation by the sea. As a result, broad FRE values were constructed using values from previous studies undertaken on the European continent; a minimum of 200 years (following (Meadows et al. 2015)) and a maximum of 1200 years (following (Meadows et al. 2018)). MRE values are also not available for the British Isles during the Late Pleistocene. As a result, a weighted average MRE value (ΔR = -191 ± 47) was calculated from around the Irish Sea (Marine Reservoir Correction Database, http://calib.org/marine/).

OxCal age model construction

The radiocarbon dates of the human remains and humanly modified faunal remains from both Gough's Cave and Kendrick's Cave were modelled using the Bayesian statistical program OxCal (version 4.4, (Bronk Ramsey 2021)) applying the IntCal20 calibration curve (Reimer et al. 2020) (Supplementary Table 7). Prior information related to the stratigraphy of the archaeological remains at both sites was gathered (Jacobi and Higham 2011) and incorporated into the model as a *Phase (Bronk Ramsey 2008)* linked to proposed human activity. A *Boundary* was placed at the beginning and end of the *Phase* to provide probability distributions for the arrival and proposed abandonment at each site (Bronk Ramsey 2008). An *Outlier_Model* was also applied to each radiocarbon date with a 5% prior probability that the date is a statistical outlier (Bronk Ramsey 2009).

Due to the stable isotope data from Kendrick's Cave indicating human consumption of terrestrial, freshwater and marine food groups, the *Mix_Curves* function was used (Christopher Bronk Ramsey 2001) with IntCal20 (Reimer et al. 2020), Marine20 curve (Heaton et al. 2020), and manually defined FRE (see above) as a broad *Uniformed* distribution (called "River", (Bronk Ramsey 2008)). A *Difference* query was also used to statistically investigate if there was a significant difference between the end of the human occupations of Gough's and age of the human remains at Kendrick's Cave (Bronk Ramsey 2001) (see Supplementary Table 7).

One *Phase* was defined for Gough's Cave using previous archaeological excavation information. Of the seventeen radiocarbon dates for the sites, two of these (OxA-18067 and OxA-17832) were removed from the model due to low agreement indices in the age model generated here (15.0% and 43.5% respectively). (Jacobi and Higham 2009/9) had previously included OxA-17832 in the age model for the site even though there was low agreement.

However, with the inclusion of *Outlier* analysis, OxA-17832 was downweighed to <5% in our model, further indicating the date as an outlier. As a result, OxA-17832 was subsequently removed to improve the overall uncertainty for Gough's Cave.

At Kendrick's Cave, two *Phases* separated by a lime breccia boundary were defined following stratigraphic information provided by archaeological excavations (information found in Jacobi and Higham 2009; Jacobi and Higham 2011). No radiocarbon dates were removed as a result of agreement indices and the *Outlier* results. When calibrating the ages for the human individuals, including the dietary reservoir effects results in younger dates than when no reservoir effect is applied (Supplementary Figure 2). This highlights the importance of incorporating stable isotope investigations and reservoir effects when investigating the timing of human occupations using human skeletal remains. However, these age estimates are not entirely robust due to the large uncertainties associated with the FRUITS model for each reconstructed dietary component percentage and the estimated MRE and FRE. Additional refinement of these dietary uncertainties and further insight could be achieved in future (e.g. via compound specific isotope analysis (CSIA) or $\delta^{34}S$), which would subsequently improve the age models.

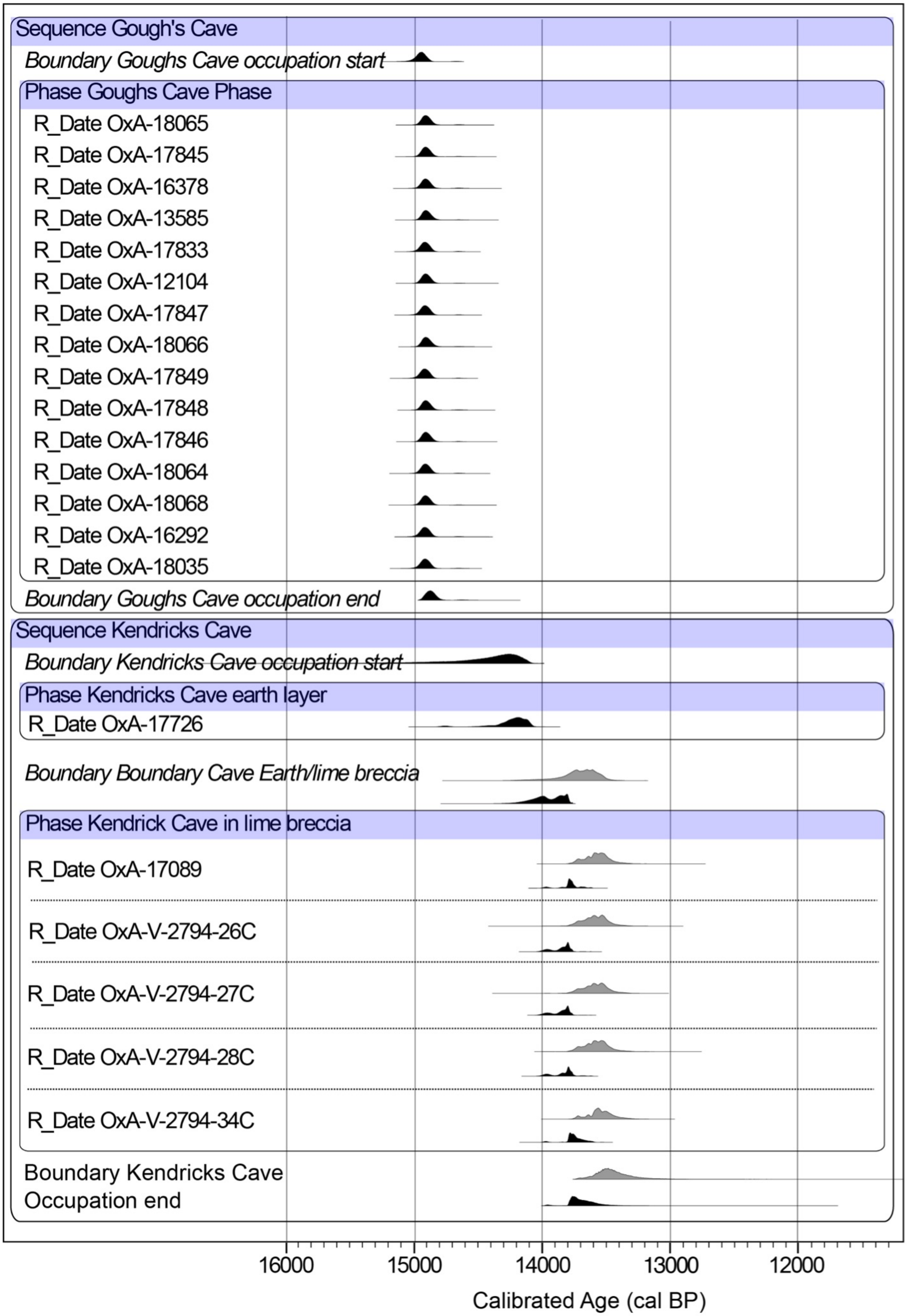
Sequence Gough's Cave
Boundary Goughs Cave occupation start
Phase Goughs Cave Phase
R_Date OxA-18065
R_Date OxA-17845
R_Date OxA-16378
R_Date OxA-13585
R_Date OxA-17833
R_Date OxA-12104
R_Date OxA-17847
R_Date OxA-18066
R_Date OxA-17849
R_Date OxA-17848
R_Date OxA-17846
R_Date OxA-18064
R_Date OxA-18068
R_Date OxA-16292
R_Date OxA-18035
Boundary Goughs Cave occupation end
Sequence Kendricks Cave
Boundary Kendricks Cave occupation start
Phase Kendricks Cave earth layer
R_Date OxA-17726
Boundary Boundary Cave Earth/lime breccia
Phase Kendrick Cave in lime breccia
R_Date OxA-17089
R_Date OxA-V-2794-26C
R_Date OxA-V-2794-27C
R_Date OxA-V-2794-28C
R_Date OxA-V-2794-34C
Boundary Kendricks Cave
Occupation end
16000
15000
14000
13000
12000
Calibrated Age (cal BP)

Supplementary Figure 2: Calibrated radiocarbon ages from Gough's Cave and Kendrick's Cave using OxCal *Mix_Curves* function and IntCal20. For the Kendrick's Cave human remains, calibrated radiocarbon dates are based on diet reconstructions produced by using FRUITS modelling with local marine reservoir effect (MRE) and variable freshwater reservoir effects (FRE). FRE is based on different magnitudes of FRE from previous research from Europe (Meadows et al. 2015, 2018). Date ranges in black indicate no MRE or FRE is applied to Kendrick's Cave human remains (i.e. these samples can not be older than this date). Date ranges in grey indicate MRE and FRE is applied (see above) to Kendrick's Cave human remains, with ages given the most likely date for the remains.

Final OxCal model

The CQL Code for the model is as follows, and the output can be found in Supplementary Table 7:

```
Plot("Goughs Cave")
 {
  Curve("IntCal20","IntCal20.14c");
  Delta_R("River",U(200,1200));
  Curve("Marine20","Marine20.14c");
  Delta_R("LocalMarine",-191,47);
  Curve("=IntCal20");
  Outlier_Model("General",T(5),U(0,4),"t");
  Sequence("Gough's Cave")
  {
   Boundary("Goughs Cave occupation start");
   Phase("Goughs Cave Phase")
   {
    R_Date("OxA-18065", 12490, 55)
    {
     Outlier(0.05);
    };
    R_Date("OxA-17845", 12500, 50)
    {
     Outlier(0.05);
    };
    R_Date("OxA-16378", 12515, 50)
    {
     Outlier(0.05);
    };
    R_Date("OxA-13585", 12440, 55)
    {
```

```
 Outlier(0.05);
};
R_Date("OxA-17833", 12570, 45)
{
 Outlier(0.05);
};
R_Date("OxA-12104", 12495, 50)
{
 Outlier(0.05);
};
R_Date("OxA-17847", 12565, 50)
{
 Outlier(0.05);
};
R_Date("OxA-18066", 12440, 55)
{
 Outlier(0.05);
};
R_Date("OxA-17849", 12590, 50)
{
 Outlier(0.05);
};
R_Date("OxA-17848", 12485, 50)
{
 Outlier(0.05);
};
R_Date("OxA-17846", 12470, 55)
{
 Outlier(0.05);
};
R_Date("OxA-18064", 12535, 55)
{
 Outlier(0.05);
};
R_Date("OxA-18068", 12520, 55)
{
 Outlier(0.05);
};
R_Date("OxA-16292", 12585, 55)
{
 Outlier(0.05);
};
R_Date("OxA-18035", 12600, 80)
{
```

```
   Outlier(0.05);
  };
  Span("Goughs cave occupation");
  Interval("Goughs cave occupationInt");
 };
 Boundary("Goughs Cave occupation end");
};
Sequence("Kendricks Cave")
{
 Boundary("Kendricks Cave occupation start");
 Phase("Kendricks Cave earth layer")
 {
  //Terrestrial Bovid
  R_Date("OxA-17726", 12310, 50)
  {
   Outlier(0.05);
  };
 };
 Boundary("Boundary Cave Earth/lime breccia");
 Phase("Kendrick Cave in lime breccia")
 {
  Mix_Curve("FishOxA-V-2794-26C","River","LocalMarine",62,26);
  Mix_Curve("MixOxA-V-2794-26C","IntCal20","FishOxA-V-2794-26C",64,20);
  R_Date("OxA-V-2794-26C",11980,50)
  {
   Outlier(0.05);
  };
  Mix_Curve("FishOxA-V-2794-27C","River","LocalMarine",61,28);
  Mix_Curve("MixOxA-V-2794-27C","IntCal20","FishOxA-V-2794-27C",57,19);
  R_Date("OxA-V-2794-27C",11990,50)
  {
   Outlier(0.05);
  };
  Mix_Curve("FishOxA-V-2794-28C","River","LocalMarine",63,30);
  Mix_Curve("MixOxA-V-2794-28C","IntCal20","FishOxA-V-2794-28C",56,19);
  R_Date("OxA-V-2794-28C",11950,50)
  {
   Outlier(0.05);
  };
  Mix_Curve("FishOxA-V-2794-34C","River","LocalMarine",59,29);
  Mix_Curve("MixOxA-V-2794-34C","IntCal20","FishOxA-V-2794-34C",54,19);
  R_Date("OxA-V-2794-34C",11830,50)
  {
   Outlier(0.05);
```

```
    };
    Mix_Curve("FishOxA-17089","River","LocalMarine",51,30);
    Mix_Curve("MixOxA-17089","IntCal20","FishOxA-17089",47,18);
    R_Date("OxA-17089",11905,50)
    {
     Outlier(0.05);
    };
    Span("Kendricks cave occupation");
    Interval("Kendricks cave occupationInt");
   };
   Boundary("Kendricks Cave occupation end");
  };
  Difference("Difference","Goughs Cave occupation end", "Kendricks Cave occupation start");
  Difference("Differencehumans","Goughs Cave occupation end", "Boundary Cave Earth/lime
breccia");
 };
```

aDNA Methods

Drilling of samples was undertaken in the dedicated ancient DNA laboratory at the Natural History Museum, London. Between 0.027-0.030g of bone or cementum powder was finely drilled from each specimen using a rotary dental drill at low speed.

Ancient DNA extractions and NGS library builds were undertaken in the dedicated ancient DNA laboratory at the Natural History Museum, London. DNA was extracted using the protocol outlined by Dabney et al. (2013), designed specifically for short DNA fragments, but replaced the Zymo-Spin V column binding apparatus with a high pure extender assembly from the High Pure Viral Nucleic Acid Large Volume Kit (Roche 05114403001). Library preparations followed a modified version of the (Meyer and Kircher 2010) protocol. The samples were also subjected to the partial uracil–DNA–glycosylase treatment described in (Rohland et al. 2015). Modifications to the library building protocol (Meyer and Kircher 2010) were that the initial DNA fragmentation step was not required, and all clean-up steps used MinElute PCR purification kits (Qiagen). The index PCR step included double indexing (Kircher, Sawyer, and Meyer 2012), the polymerase AmpliTaq Gold, and the addition of 0.4mg/mL BSA. The indexing PCR was set for 20 cycles with three PCR reactions conducted per library. Libraries were sequenced on a HiSeq4000 platform (100 bp paired-end) at The Francis Crick Institute, London. Sequencing statistics can be found in Supplementary Table 8.

Authenticity of results

All stages of aDNA analysis, from initial sampling and drilling of the material through to library amplification set up, were undertaken in the dedicated ancient DNA laboratory at the Natural

History Museum, London. Standard precautions to avoid contamination were undertaken, including the wearing of coveralls, masks, visors, dedicated lab footwear and double gloves. Working surfaces and all laboratory equipment were frequently cleaned with sodium hypocholrite (bleach) or a DNA degrading detergent (DNA AWAY), and subsequently ultraviolet-irradiated.

Contamination was further controlled and estimated by the sequencing of negative controls, and estimation of molecular damage and sequence length of generated reads. Furthermore, the sequence reads show damage patterns consistent with ancient DNA and partial uracil–DNA–glycosylase treatment (Supplementary Figures 3 and 4).

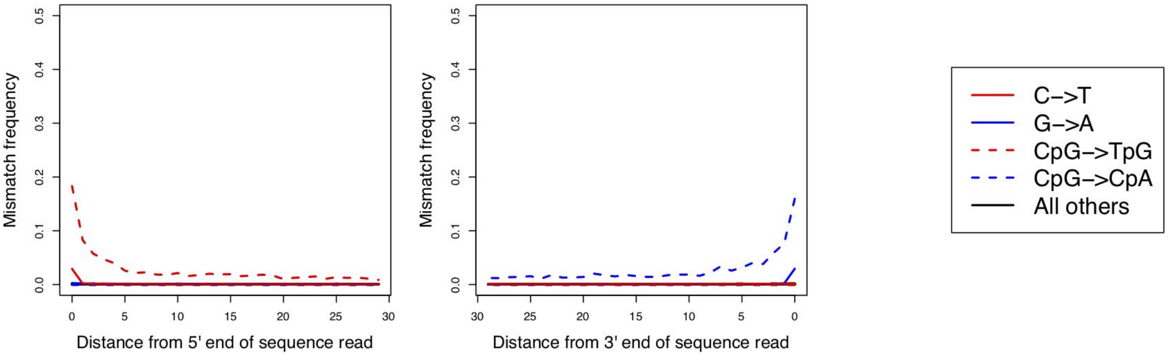

Supplementary Figure 3: aDNA damage plot for Gough’s Cave individual indicating DNA damage, generated using PMDtools (Skoglund et al. 2014)

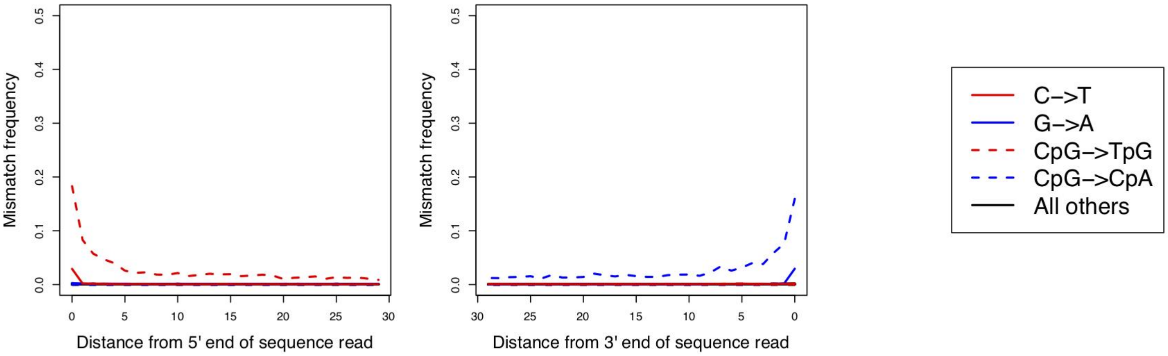

Supplementary Figure 4: aDNA damage plot for Kendrick’s Cave individual indicating DNA damage, generated using PMDtools (Skoglund et al. 2014)

Sex Identification

Genetic sex identification was undertaken using a method designed for low-coverage aDNA data (Skoglund et al. 2013), which determines the ratio of sequences aligning to the X and Y

chromosomes. This indicated that the individual from Gough's Cave is female, and the individual from Kendrick's Cave is male (Supplementary Table 2).

Sample	Nseqs	NchrY+NchrX	NchrY	R_y	SE	95% CI	Genetic sex assignment
Kendrick's Cave (SC112)	19,991,661	576,371	45,639	0.0792	0.0004	0.0785-0.0799	XY
Gough's Cave (SB392)	22,533,515	1,092,828	4,983	0.0046	0.0001	0.0044-0.0047	XX

Supplementary Table 2: Genetic sex identification data from the Kendrick's Cave and Gough's Cave individuals

Mitochondrial DNA (mtDNA) reconstruction, contamination estimates and haplogroup assignment

In order to reconstruct mitochondrial genomes of the two individuals, we re-aligned the shotgun data to the revised Cambridge reference sequence (rCRS) (Andrews et al. 1999) using Burrows Wheeler Aligner (BWA) (Li and Durbin 2010). Since BWA does not successfully align DNA fragments at the beginning and the end of a circular genome, we added 1,000 base pairs (bp) from the beginning of rCRS mitochondrial genome to its end in order to get equal coverage of the fragments across the mtDNA (Prüfer et al. 2014). Only mapped DNA fragments with a length of at least 35 bp and a mapping quality of at least 25 were retained for downstream analyses (L ≥ 35bp, MQ ≥ 25). We recovered 15,497 and 9,702 unique mtDNA fragments for the Gough's and Kendrick's Cave individuals, resulting in 53.8-fold and 34.8-fold average coverage respectively. We reconstructed their full mitochondrial genomes once by calling a consensus base at each position along the genome that was covered by at least three DNA fragments and where at least 67% of fragments had an identical base (Meyer et al. 2014), and once by using *schmutzi* (Renaud et al. 2015). The mitochondrial genomes reconstructed with both methods were identical to each other.

Using *schmutzi* (Renaud et al. 2015), an iterative probabilistic method, we estimated the proportion of present-day human DNA contamination among mtDNA fragments of Gough's and Kendrick's Cave individuals (parameters: "—notusepredC —uselength") to be 0.5% (95% confidence intervals (CI): 0-1%) and 2% (95% CI: 1-3%) respectively.

The haplogroups of the reconstructed mtDNA genomes were identified using HaploGrep (Kloss-Brandstätter et al. 2011) and the Phylotree database (build 17). The Gough's Cave individual

carries the substitutions that define the haplogroup U8a (0.97 posterior support), and the Kendrick's Cave individual the substitutions that define the haplogroup U5a2 (1.0 posterior support).

mtDNA phylogeny

The reconstructed mtDNA genomes of the Gough's and Kendrick's Cave individuals were aligned to the mtDNA genomes of 54 present-day (Green et al. 2008) and 52 ancient modern humans of known ages (Krause et al. 2010; Benazzi et al. 2015; Fu, Mittnik, et al. 2013; Fu, Meyer, et al. 2013; Fu et al. 2014, 2015; Gilbert et al. 2008; Posth et al. 2016; Hublin et al. 2020) which served as calibration points for tip dating, using MAFFT v7.271 (Katoh and Standley 2013). We used the Vindija 33.16 Neandertal mtDNA genome (Green et al. 2008) as an outgroup to root the tree. The best-fitting substitution model for this dataset determined using jModelTest2 (Darriba et al. 2012) was Tamura-Nei 93 with a fixed fraction of invariable sites and gamma distributed rates (TN93+I+G). We investigated the models of rate variation and tree priors following the steps detailed in (Posth et al. 2016; Hublin et al. 2020). We determined the strict clock model and Bayesian skyline to be the best fit to the data following a marginal likelihood estimation (MLE) (Baele et al. 2012) analysis for model comparison and best support assessment. We estimated the age of Gough's Cave individual to be 15,430 years BP (95% highest posterior density (HPD): 9,770-22,330) and Kendrick's Cave individual to be 13,460 years BP (95% HPD: 6,470-22,900). The resulting tree (Supplementary Figure 5) was visualised using FigTree (version: v1.4.2) (http://tree.bio.ed.ac.uk/software/figtree/).

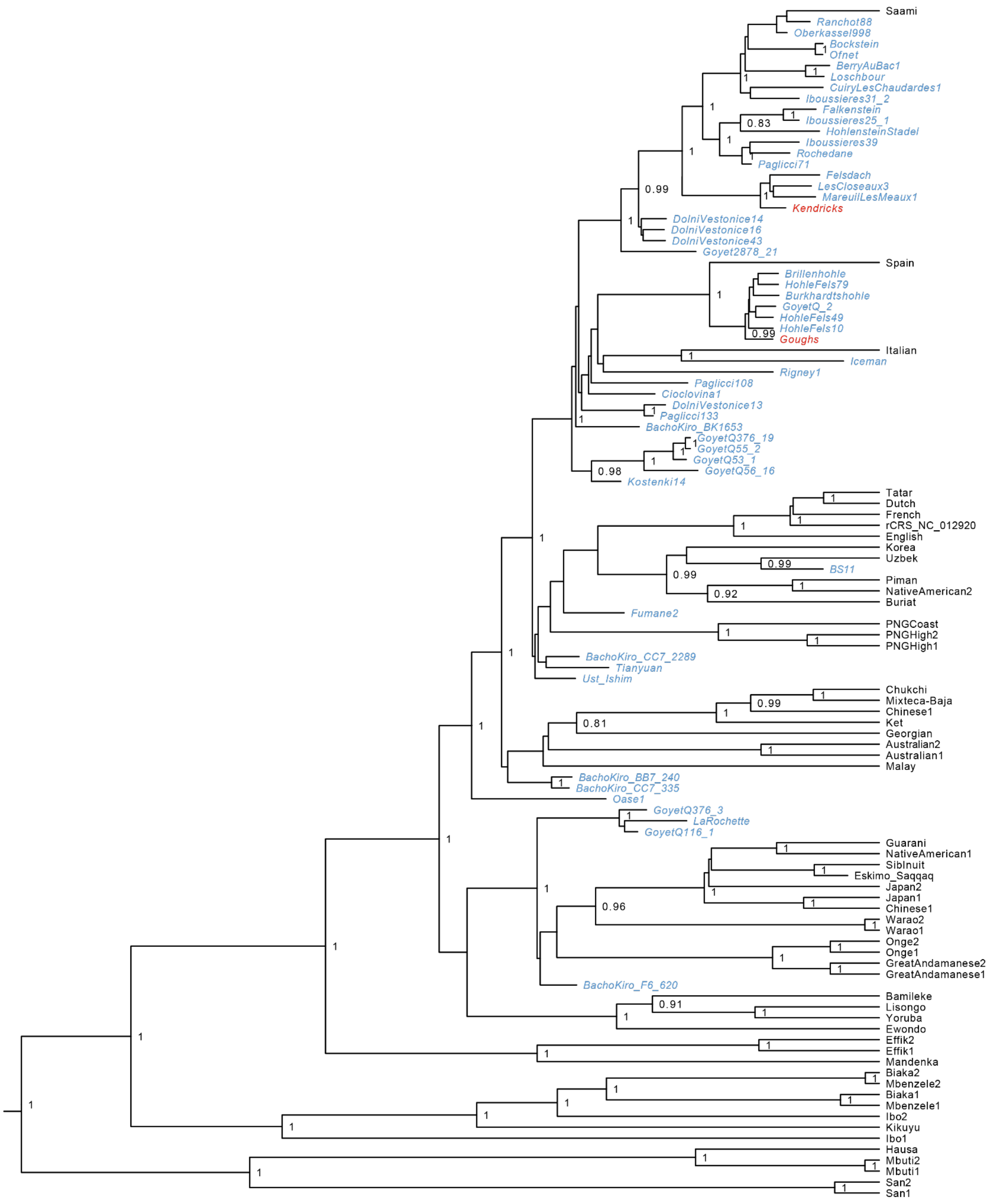

Supplementary Figure 5: Bayesian phylogenetic tree relating Gough's Cave and Kendrick's Cave mtDNA genomes to 52 radiocarbon dated ancient humans and 54 present-day humans. The Gough's Cave and Kendrick's Cave mtDNA are highlighted in red and other ancient humans are highlighted in light blue. Present-day human mtDNA is in black. Posterior probabilities are denoted above the branches and the mtDNA of Vindija 33.16 was used to root the tree (not shown)

Nuclear DNA content – overlap with the "1240k" dataset and random read sampling

Using BEDtools (Quinlan and Hall 2010) (version 2.23.0) we intersected the shotgun data generated from the Gough's Cave and Kendrick's Cave individuals with a combined 1,233,013 single nucleotide polymorphisms (SNPs) from Panel 1 in (Haak et al. 2015) ("390k") and Panel 2 from (Fu et al. 2015) ("840k"), known together as the "1240k" SNP Panel. We performed random read sampling using bam-caller (https://github.com/bodkan/bam-caller, version: 0.1) by picking a base at each position overlapping the "1240k" SNP Panel that was covered by at least one DNA fragment longer than 35 bp with a mapping quality of at least 25 (L≥35bp, MQ≥25). We masked the first two and the last two bases at the alignment start and end in each DNA fragment to mitigate the effect of deamination-derived substitutions on downstream analyses, amounting to 506,151 (41.05%) and 476,347 (38.63%) of informative SNPs for the Gough's and Kendrick's Cave individuals. We further combined the SNPs of the two individuals with the genotypes of 5,225 ancient and 3,720 present-day humans of the Allen Ancient DNA resource (release 20th January 2021, version 44.3, https://reich.hms.harvard.edu/allen-ancient-dna-resource-aadr-downloadable-genotypes-present-day-and-ancient-dna-data).

Principal Component Analysis (PCA)

As an assessment of the genetic affinities of the two individuals, we carried out Principal Component Analysis (PCA) using *smartpca* from the EIGENSOFT package (Patterson, Price, and Reich 2006; Price et al. 2006). We used 1,087 present-day humans from West Eurasia genotyped on 597,573 SNPs of the Affymetrix Human Origins array (Patterson et al. 2012) (retrieved from the Allen Ancient DNA resource, release 20th January 2021, version 44.3, https://reich.hms.harvard.edu/allen-ancient-dna-resource-aadr-downloadable-genotypes-present-day-and-ancient-dna-data) to estimate the eigenvectors. We then projected 168 ancient hunter gatherers from west Eurasia and north Africa older than ~7,000 years cal. BP (Supplementary Table 3) and which have more than 30,000 informative SNPs onto the plane defined by these eigenvectors (Figure 2, Main Manuscript) using the 'lsqproject' option in *smartpca* (Patterson, Price, and Reich 2006).

F-statistics and population relationships of Gough's and Kendrick's individuals

F-statistics and D-statistics were calculated using ADMIXTOOLS (Patterson et al. 2012)(version 5.1) as implemented in the R package *admixr (Petr, Vernot, and Kelso, n.d.)* (version 0.9.1), computing standard errors using a Weighted Block Jackknife (Busing, Meijer, and Van Der Leeden 1999; Patterson et al. 2012) across all autosomes with equally sized blocks of 5 million base pairs (5 Mb). We computed the statistics of the form $f_3(X, Y; Mbuti)$, which measures the

levels of genetic drift shared between populations X and Y since their separation from an outgroup, and 263 present-day humans from the Simons Genome Diversity Project (SGDP) (Mallick et al. 2016). We find that both Gough's and Kendrick's Cave individuals share most drift with present-day West Eurasians (Supplementary Figures 6 and 7).

When restricting the f_3-statistics analyses to ancient hunter gatherers from west Eurasia and north Africa and which are older than 7,000 years cal. BP in the form f_3*(ancient1, ancient2; Mbuti)*, we find that Gough's Cave individual shares most drift with the members of previously defined "GoyetQ2" genetic cluster (Villalba-Mouco et al. 2019), and Kendrick's Cave individual shares most drift with the members of "Villabruna" genetic cluster (Fu et al. 2016) (Figure 3, Main Manuscript).

In order to investigate the relationships of the two individuals to other ancient humans in more detail, we used D-statistics in the form *D(ancient1, ancient2; Gough's/Kendrick's, Mbuti)*, *D(Gough's/Kendrick's, ancient1; ancient2, Mbuti)* and *D(Gough's, Kendrick's; ancient, Mbuti)* (Supplementary Figures 8-11).

qpAdm and qpWave modelling

We used *qpAdm* from ADMIXTOOLS (version 5.1) (Patterson et al. 2012) to model the ancestry of the Gough's Cave and Kendrick's Cave individuals with a default "useallsnps: NO" option. Guided by the results of the D-statistics and F-statistics, we used the ~15,000-year-old GoyetQ2 individual from Belgium (Fu et al. 2016; Villalba-Mouco et al. 2019) and the ~14,000-year-old Villabruna individual from Italy (Fu et al. 2016) as source populations, and the following set of reference populations (Villalba-Mouco et al. 2019): Ust'Ishim (Fu et al. 2014), GoyetQ116-1 (Fu et al. 2016), Mal'ta 1 (Raghavan et al. 2014), Natufian (Lazaridis et al. 2016), Koros (Lipson et al. 2017), Mota (Gallego Llorente et al. 2015), Onge, Han, Papuan, Karitiana and Mbuti (Mallick et al. 2016). We explored both single-source (*qpWave*) and two-source models using a 'rotating model' approach (Skoglund et al. 2017; Harney et al. 2020). Models were accepted when the P-value was above the significance cut-off of 0.05 (Patterson et al. 2012; Harney et al. 2020), and the lowest rank models were chosen when a model with a higher rank could also be used to explain the data.

Since both the Gough's Cave and Kendrick's Cave individuals could be modelled as single-source 'GoyetQ2' and 'Villabruna' (Figure 4 and Supplementary Figure 12) respectively, we investigated whether a lower-rank model is preferred due to the limited amount of data we have for these two individuals. To do so, we down-sampled the ~10,000-year-old Cheddar Man from Britain to the same number of sites as in Gough's and Kendrick's Cave individuals. Even with the reduced number of informative SNPs, single-source models are rejected for Cheddar Man (P-value: 0.004).

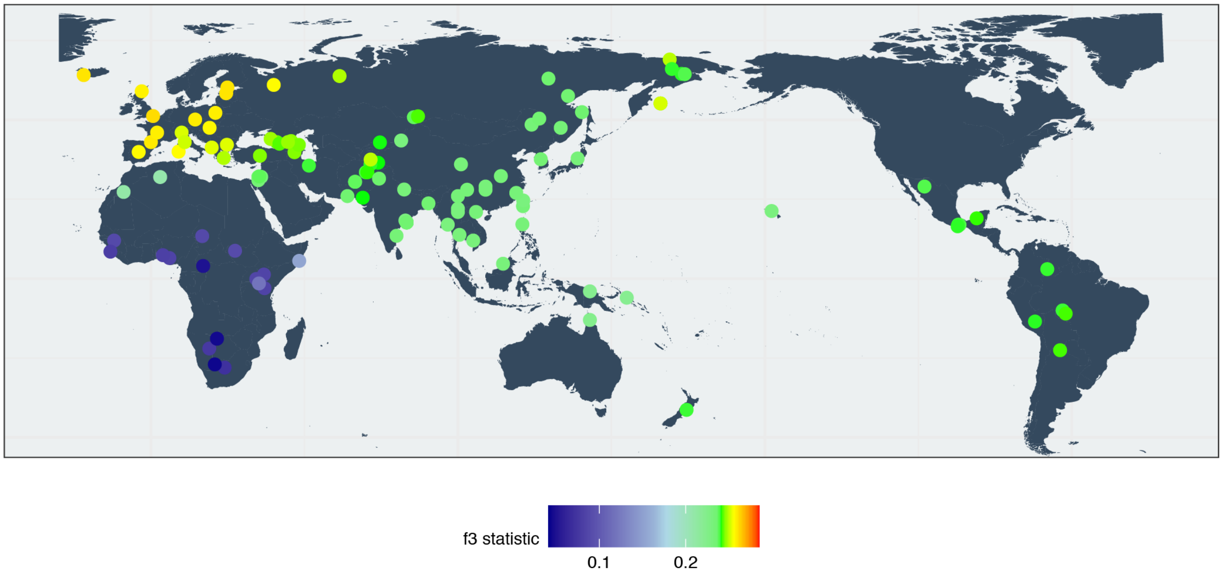

Supplementary Figure 6: Outgroup *f3*-statistics of the form *f3(Gough's Cave, present-day population; Mbuti)* measuring the amount of shared genetic drift between the Gough's individual and a range of present-day human populations from the Simons Genome Diversity Project (SGDP). Higher *f3*-values are indicated with warmer colours and correspond to higher amounts of shared genetic drift between the Gough's Cave individual and a given present-day human population. Heatmap scale is consistent with the one for Kendrick's Cave individual in Supplementary Figure 7

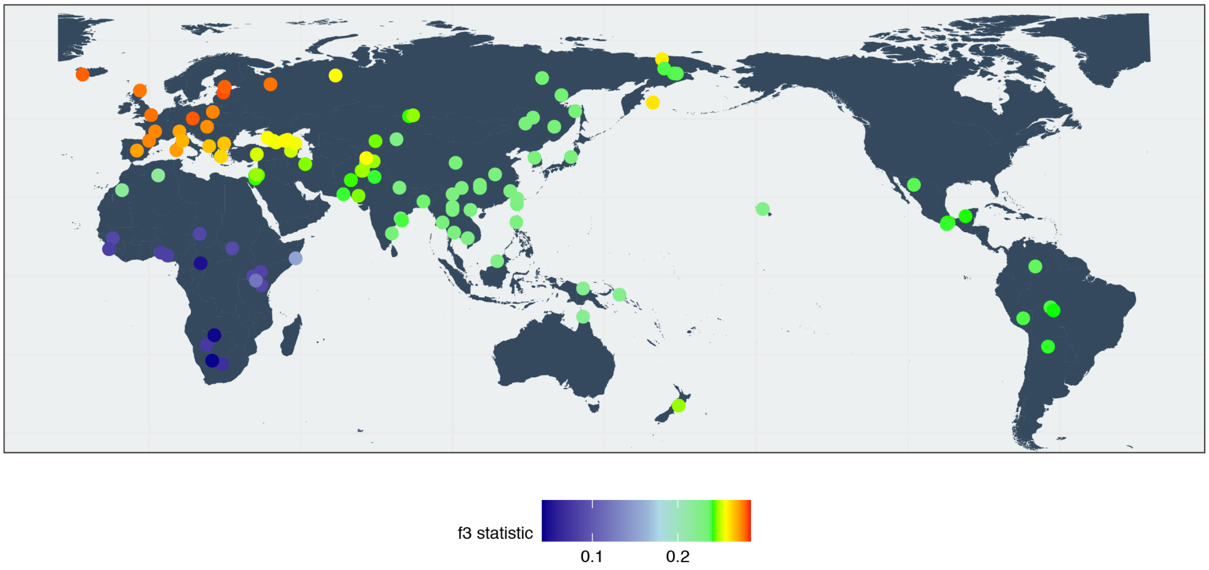

Supplementary Figure 7: Outgroup *f3*-statistics of the form *f3(Kendrick's Cave, present-day population; Mbuti)* measuring the amount of shared genetic drift between the Kendrick's individual and a range of present-day human populations from the Simons Genome Diversity Project (SGDP). Higher *f3*-values are indicated with warmer colours and correspond to higher amounts of shared genetic drift between the Kendrick's Cave individual and a given present-day human population. Heatmap scale is consistent with the one for Gough's Cave individual in Supplementary Figure 6

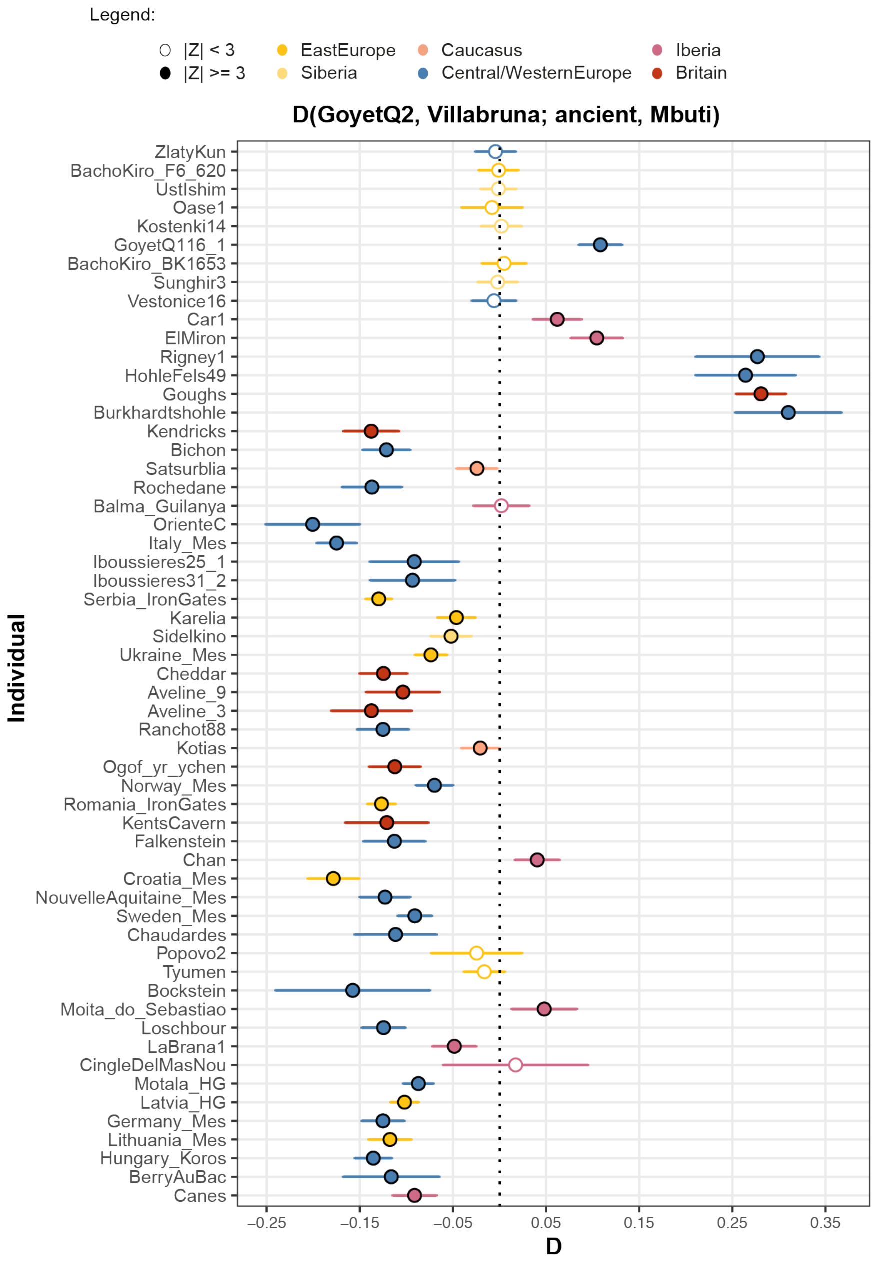

Supplementary Figure 8: *D(GoyetQ2, Villabruna; ancient, Mbuti)* measuring the proportion of allele sharing between a given ancient individual (ordered chronologically from the oldest to the youngest on the y-axis, n=57) with the ~15,000-year-old Goyet Q2 individual from Belgium and the ~14,000-year-old Villabruna from Italy. D-values denoted as circles on the x-axis were calculated using ADMIXTOOLS as implemented in admixr. Filled-in circles correspond to a significant Z-score or |Z| ≥ 3, and open circles indicate a non-significant Z-score or |Z| < 3.

Standard errors (SE) were calculated using Weighted Block Jackknife and a block size of 5 Mb.

Whiskers correspond to 3SE

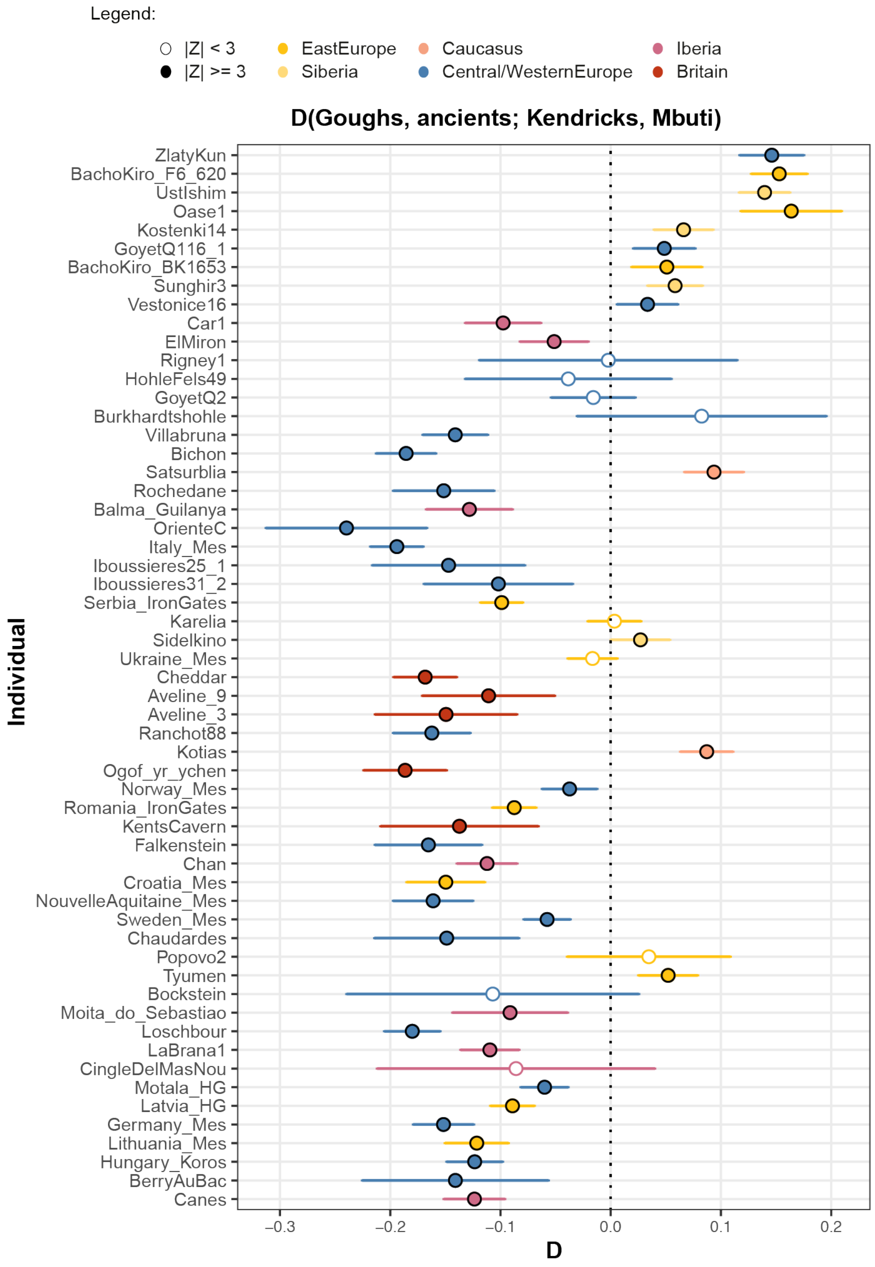

Supplementary Figure 9: *D(Gough's Cave, ancient; Kendrick's Cave, Mbuti).* D-values denoted as circles on the x-axis were calculated using ADMIXTOOLS as implemented in admixr. Ancient individuals (n=57) on the y-axis are ordered chronologically from the oldest to the youngest. Filled-in circles correspond to a significant Z-score or |Z| ≥ 3, and open circles indicate a non-significant Z-score or |Z| < 3. Standard errors (SE) were calculated using Weighted Block Jackknife and a block size of 5 Mb. Whiskers correspond to 3SE

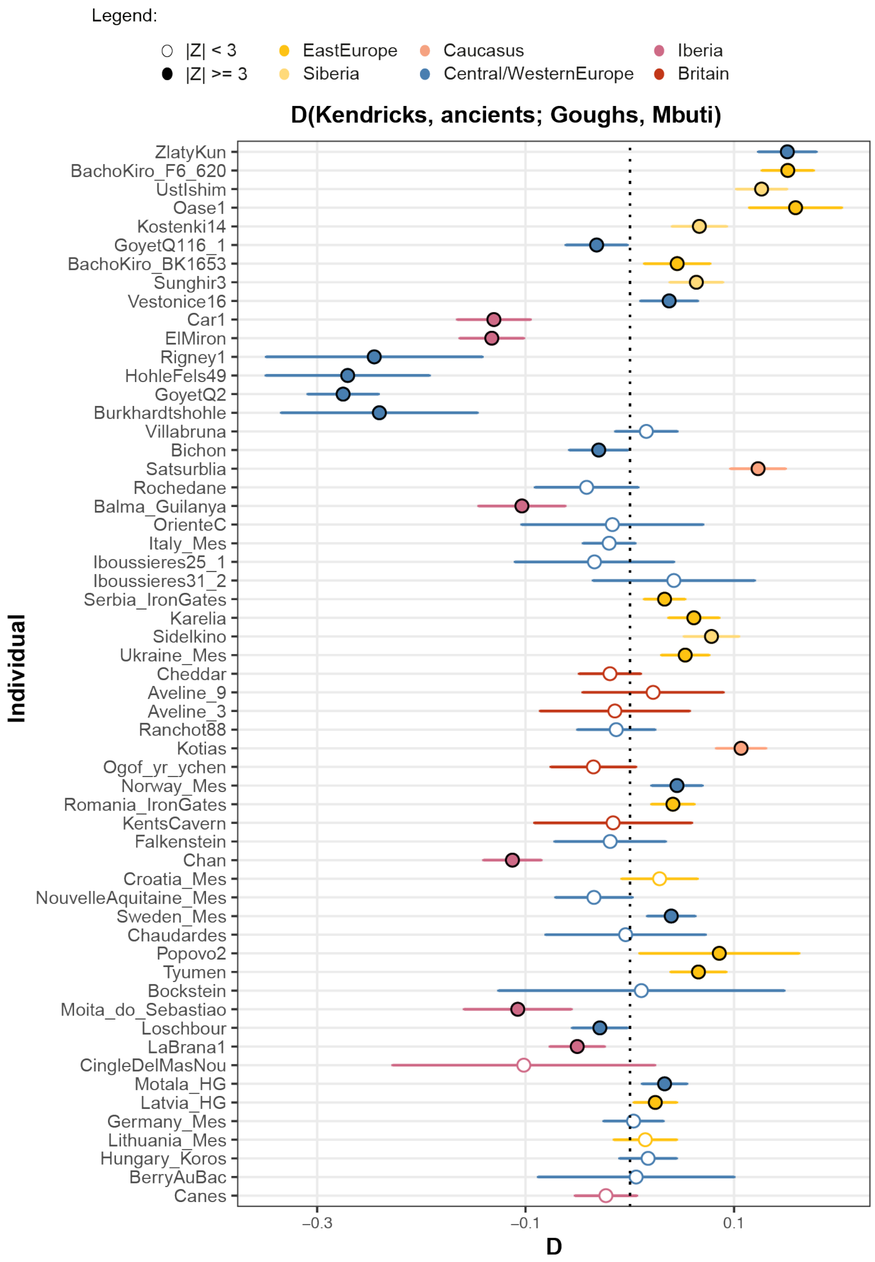

Supplementary Figure 10: *D(Kendrick's Cave, ancient; Gough's Cave, Mbuti).* D-values denoted as circles on the x-axis were calculated using ADMIXTOOLS as implemented in admixr. Ancient individuals (n=57) on the y-axis are ordered chronologically from the oldest to the youngest. Filled-in circles correspond to a significant Z-score or |Z| ≥ 3, and open circles indicate a non-significant Z-score or |Z| < 3. Standard errors (SE) were calculated using Weighted Block Jackknife and a block size of 5 Mb. Whiskers correspond to 3SE

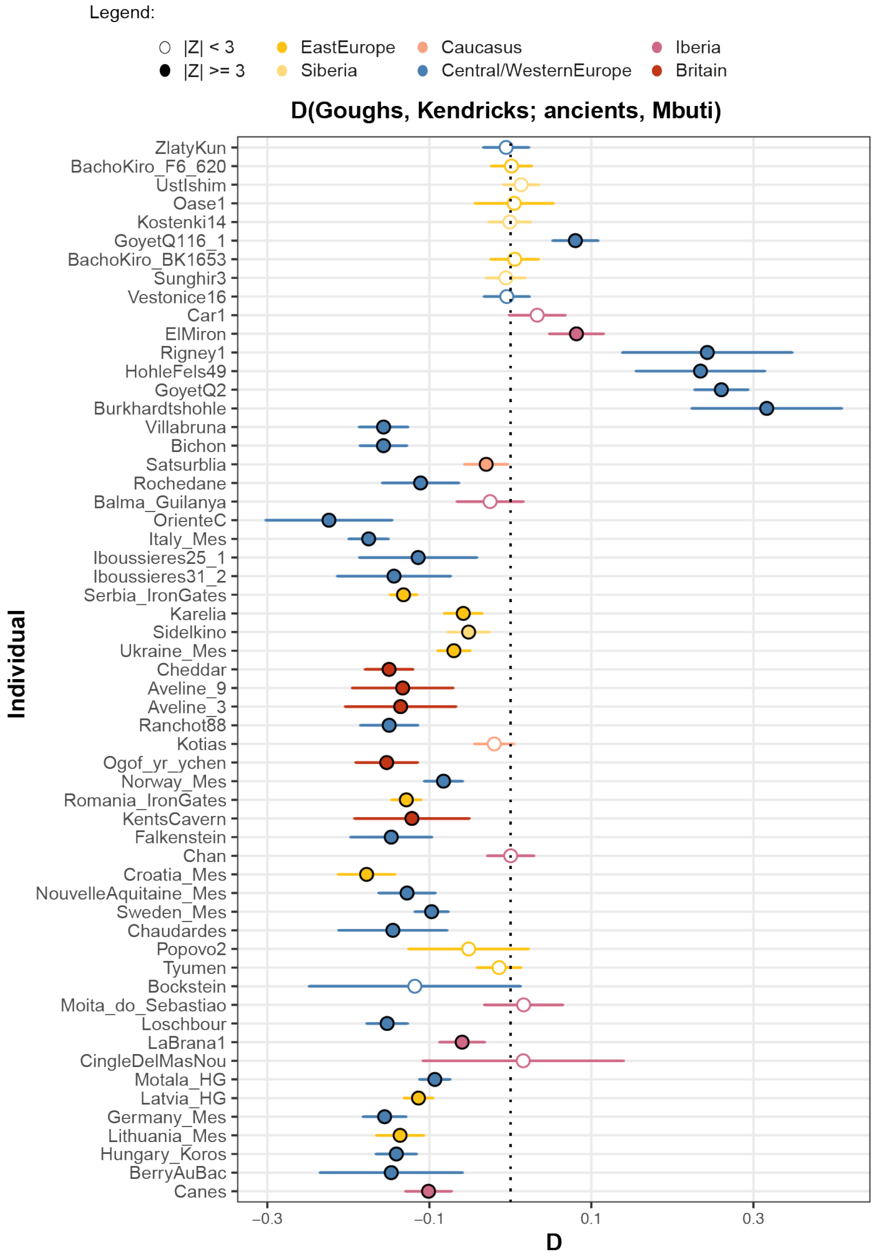

Supplementary Figure 11: *D(Gough's Cave, Kendrick's Cave; ancient, Mbuti).* D-values denoted as circles on the x-axis were calculated using ADMIXTOOLS as implemented in admixr. Ancient individuals (n=57) on the y-axis are ordered chronologically from the oldest to the youngest. Filled-in circles correspond to a significant Z-score or |Z| ≥ 3, and open circles indicate a non-significant Z-score or |Z| < 3. Standard errors (SE) were calculated using Weighted Block Jackknife and a block size of 5 Mb. Whiskers correspond to 3SE

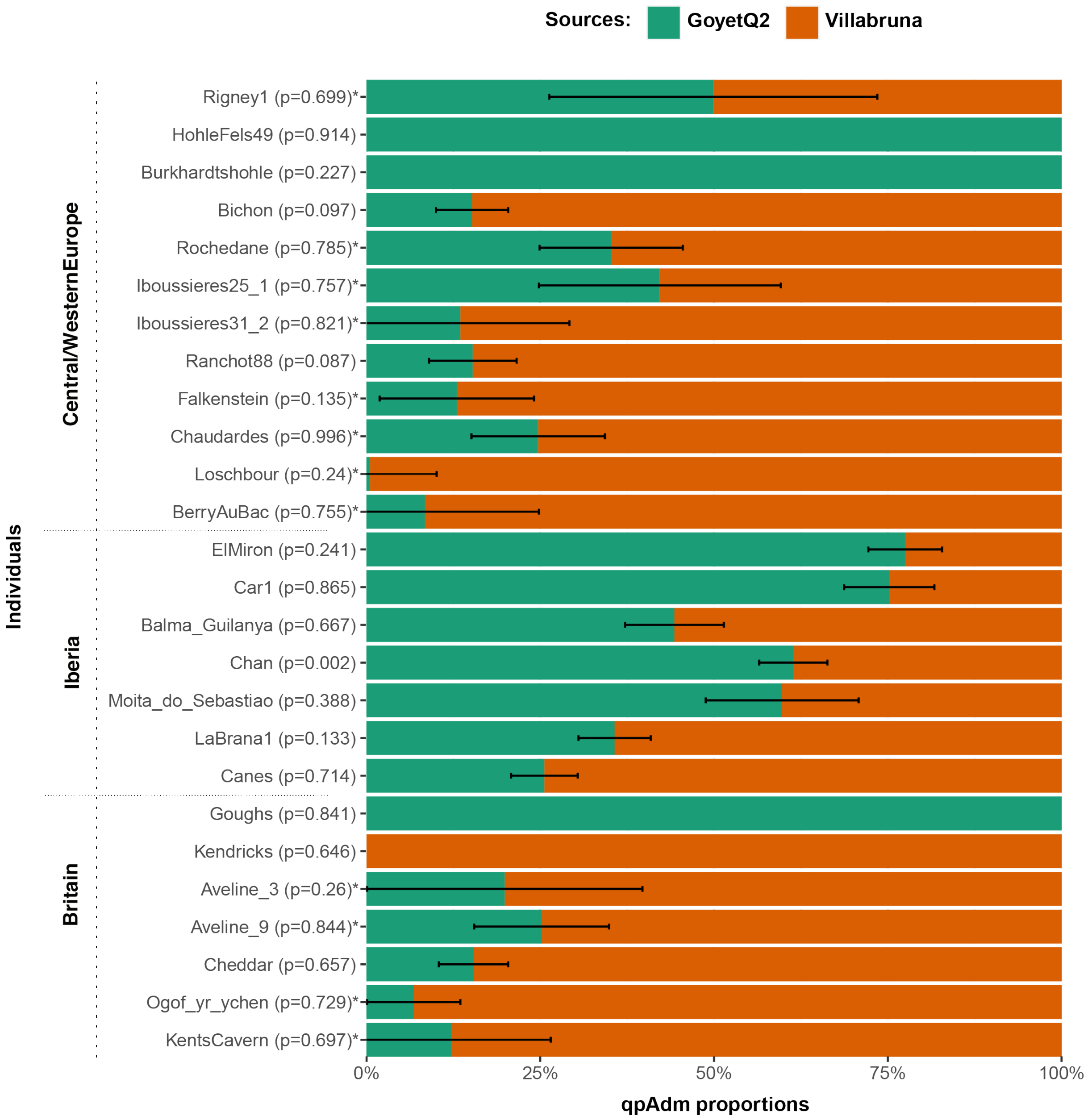

Supplementary Figure 12: Modelling West Eurasian HG individuals (n=26) as having a single-source or a two-source ancestry with qpAdm, by taking into account if the two-source (higher-rank) models fit for individuals where a single-source model is sufficient to explain the data. The ~15,000-year-old GoyetQ2 individual from Belgium and the ~14,000-year-old Villabruna from Italy were used as potential sources. Within each geographical area (Central/Western Europe; Iberia; Britain) ancient individuals are ordered chronologically from the oldest (on top) to the youngest (on bottom). The * next to a given individual indicates that a single-source (lower-rank) model fits the data as well. Standard errors (SE) were calculated using Weighted Block Jackknife and a block size of 5 Mb. SE here indicates +-1SE

Reference List

Andrews, Richard M., Iwona Kubacka, Patrick F. Chinnery, Robert N. Lightowlers, Douglass M. Turnbull, and Neil Howell. 1999. "Reanalysis and Revision of the Cambridge Reference Sequence for Human Mitochondrial DNA." *Nature Genetics* 23 (2): 147–147.

Baele, Guy, Philippe Lemey, Trevor Bedford, Andrew Rambaut, Marc A. Suchard, and Alexander V. Alekseyenko. 2012. "Improving the Accuracy of Demographic and Molecular Clock Model Comparison While Accommodating Phylogenetic Uncertainty." *Molecular Biology and Evolution* 29 (9): 2157–67.

Benazzi, S., V. Slon, S. Talamo, F. Negrino, M. Peresani, S. E. Bailey, S. Sawyer, et al. 2015. "Archaeology. The Makers of the Protoaurignacian and Implications for Neandertal Extinction." *Science* 348 (6236): 793–96.

Bocherens, Herve, and Dorothée Drucker. 2006. "Isotope Evidence for Paleodiet of Late Upper Paleolithic Humans in Great Britain: A Response to Richards et Al. 2005." *Journal of Human Evolution* 51: 440–42.

Brock, Fiona, Thomas Higham, Peter Ditchfield, and Christopher Bronk Ramsey. 2010. "Current Pretreatment Methods for AMS Radiocarbon Dating at the Oxford Radiocarbon Accelerator Unit (ORAU)." *Radiocarbon* 52 (1): 103–12.

Bronk Ramsey, C. 2021. "OxCal Project, Version 4.4." 2021. https://c14.arch.ox.ac.uk/oxcal/.

Bronk Ramsey, Christopher. 2001. "Development of the Radiocarbon Calibration Program." *Radiocarbon* 43 (2A): 355–63.

———. 2008. "Deposition Models for Chronological Records." *Quaternary Science Reviews* 27 (1): 42–60.

———. 2009. "Dealing with Outliers and Offsets in Radiocarbon Dating." *Radiocarbon* 51 (3): 1023–45.

Bronk Ramsey, C., Higham, T. H. F., Owen, D. C., Pike, A. W. G., & Hedges, R. E. M. 2002. "Radiocarbon dates from the Oxford AMS system: Archaeometry Datelist 31." *Archaeometry* 44(3s), 1-149

Busing, Frank Mta, Erik Meijer, and Rien Van Der Leeden. 1999. "Delete-M Jackknife for Unequal M." *Statistics and Computing* 9 (1): 3–8.

Charles R. 1989. "Incised ivory fragments and other Late Upper Palaeolithic finds from Gough's Cave, Cheddar, Somerset." *Proc Univ Bristol Spelaeol Soc.* 18: 400–408.

Currant, Andrew, and Roger Jacobi. 2001. "A Formal Mammalian Biostratigraphy for the Late Pleistocene of Britain." *Quaternary Science Reviews* 20 (16–17): 1707–16.

Currant, A. P. 1986. "The Lateglacial Mammal Fauna of Gough's Cave, Cheddar, Somerset." *Proc. Univ. Bristol Spelaeol. Soc* 17 (3): 286–304.

Dabney, Jesse, Michael Knapp, Isabelle Glocke, Marie-Theres Gansauge, Antje Weihmann, Birgit Nickel, Cristina Valdiosera, et al. 2013. "Complete Mitochondrial Genome Sequence of a Middle Pleistocene Cave Bear Reconstructed from Ultrashort DNA Fragments." *Proceedings of the National Academy of Sciences of the United States of America* 110 (39): 15758–63.

Darriba, Diego, Guillermo L. Taboada, Ramón Doallo, and David Posada. 2012. "jModelTest 2: More Models, New Heuristics and Parallel Computing." *Nature Methods* 9 (8): 772–772.

Davies, M. 1989. "Cave Archaeology in North Wales." In *Limestones and Caves of Wales*, edited by T. D. Ford, 92–101. Cambridge: Cambridge University Press.

Dawkins, W. B. 1880. "Memorandum on the Remains from the Cave at the Great Ormes Head." *Proceedings of the Liverpool Geological Society* 4: 156–59.

Eskrigge, R. A. 1879. "Notes on Human Skeletons and Traces of Human Workmanship Found in a Cave at Llandudno." *Proceedings of the Liverpool Geological Society* 4: 153–55.

Fernandes, Ricardo, Andrew R. Millard, Marek Brabec, Marie-Josée Nadeau, and Pieter Grootes. 2014. “Food Reconstruction Using Isotopic Transferred Signals (FRUITS): A Bayesian Model for Diet Reconstruction.” *PloS One* 9 (2): e87436.

Fu, Qiaomei, Mateja Hajdinjak, Oana Teodora Moldovan, Silviu Constantin, Swapan Mallick, Pontus Skoglund, Nick Patterson, et al. 2015. “An Early Modern Human from Romania with a Recent Neanderthal Ancestor.” *Nature* 524 (7564): 216–19.

Fu, Qiaomei, Heng Li, Priya Moorjani, Flora Jay, Sergey M. Slepchenko, Aleksei A. Bondarev, Philip L. F. Johnson, et al. 2014. “Genome Sequence of a 45,000-Year-Old Modern Human from Western Siberia.” *Nature* 514 (7523): 445–49.

Fu, Qiaomei, Matthias Meyer, Xing Gao, Udo Stenzel, Hernán A. Burbano, Janet Kelso, and Svante Pääbo. 2013. “DNA Analysis of an Early Modern Human from Tianyuan Cave, China.” *Proceedings of the National Academy of Sciences of the United States of America* 110 (6): 2223–27.

Fu, Qiaomei, Alissa Mittnik, Philip L. F. Johnson, Kirsten Bos, Martina Lari, Ruth Bollongino, Chengkai Sun, et al. 2013. “A Revised Timescale for Human Evolution Based on Ancient Mitochondrial Genomes.” *Current Biology: CB* 23 (7): 553–59.

Fu, Qiaomei, Cosimo Posth, Mateja Hajdinjak, Martin Petr, Swapan Mallick, Daniel Fernandes, Anja Furtwängler, et al. 2016. “The Genetic History of Ice Age Europe.” *Nature* 534 (7606): 200–205.

Gallego Llorente, M., E. R. Jones, A. Eriksson, V. Siska, K. W. Arthur, J. W. Arthur, M. C. Curtis, et al. 2015. “Ancient Ethiopian Genome Reveals Extensive Eurasian Admixture throughout the African Continent.” *Science* 350 (6262): 820–22.

Gilbert, M. Thomas P., Toomas Kivisild, Bjarne Grønnow, Pernille K. Andersen, Ene Metspalu, Maere Reidla, Erika Tamm, et al. 2008. “Paleo-Eskimo mtDNA Genome Reveals Matrilineal Discontinuity in Greenland.” *Science* 320 (5884): 1787–89.

Green, Richard E., Anna-Sapfo Malaspinas, Johannes Krause, Adrian W. Briggs, Philip L. F. Johnson, Caroline Uhler, Matthias Meyer, et al. 2008. “A Complete Neandertal Mitochondrial Genome Sequence Determined by High-Throughput Sequencing.” *Cell* 134 (3): 416–26.

Haak, Wolfgang, Iosif Lazaridis, Nick Patterson, Nadin Rohland, Swapan Mallick, Bastien Llamas, Guido Brandt, et al. 2015. “Massive Migration from the Steppe Was a Source for Indo-European Languages in Europe.” *Nature* 522 (7555): 207–11.

Harney, Éadaoin, Nick Patterson, David Reich, and John Wakeley. 2020. “Assessing the Performance of qpAdm: A Statistical Tool for Studying Population Admixture.” *bioRxiv*. https://doi.org/10.1101/2020.04.09.032664.

Heaton, Timothy J., Maarten Blaauw, Paul G. Blackwell, Christopher Bronk Ramsey, Paula J. Reimer, and E. Marian Scott. 2020. “The IntCal20 Approach to Radiocarbon Calibration Curve Construction: A New Methodology Using Bayesian Splines and Errors-in-Variables.” *Radiocarbon* 62 (4): 821–63.

Higham, T. F. G., R. M. Jacobi, and C. Bronk Ramsey. 2006. “AMS Radiocarbon Dating of Ancient Bone Using Ultrafiltration.” *Radiocarbon* 48 (2): 179–95.

Hublin, Jean-Jacques, Nikolay Sirakov, Vera Aldeias, Shara Bailey, Edouard Bard, Vincent Delvigne, Elena Endarova, et al. 2020. “Initial Upper Palaeolithic Homo Sapiens from Bacho Kiro Cave, Bulgaria.” *Nature* 581 (7808): 299–302.

Jacobi, R. M., and T. F. G. Higham. 2009. “The Early Lateglacial Re-Colonization of Britain: New Radiocarbon Evidence from Gough’s Cave, Southwest England.” *Quaternary Science Reviews* 28 (19–20): 1895–1913.

Jacobi, Roger. 2004. “The Late Upper Palaeolithic Lithic Collection from Gough’s Cave, Cheddar, Somerset and Human Use of the Cave.” *Proceedings of the Prehistoric Society* 70: 1–92.

Jacobi, Roger, and Tom Higham. 2011. “The Later Upper Palaeolithic Recolonisation of Britain:

New Results from AMS Radiocarbon Dating." In *The Ancient Human Occupation of Britain*, edited by N. M. Ashton, S. G. Lewis, and C. B. Stringer, 14:223–47. Developments in Quaternary Science. Elsevier.
Katoh, Kazutaka, and Daron M. Standley. 2013. "MAFFT Multiple Sequence Alignment Software Version 7: Improvements in Performance and Usability." *Molecular Biology and Evolution* 30 (4): 772–80.
Kircher, Martin, Susanna Sawyer, and Matthias Meyer. 2012. "Double Indexing Overcomes Inaccuracies in Multiplex Sequencing on the Illumina Platform." *Nucleic Acids Research* 40 (1): e3.
Kloss-Brandstätter, Anita, Dominic Pacher, Sebastian Schönherr, Hansi Weissensteiner, Robert Binna, Günther Specht, and Florian Kronenberg. 2011. "HaploGrep: A Fast and Reliable Algorithm for Automatic Classification of Mitochondrial DNA Haplogroups." *Human Mutation* 32 (1): 25–32.
Krause, Johannes, Adrian W. Briggs, Martin Kircher, Tomislav Maricic, Nicolas Zwyns, Anatoli Derevianko, and Svante Pääbo. 2010. "A Complete mtDNA Genome of an Early Modern Human from Kostenki, Russia." *Current Biology: CB* 20 (3): 231–36.
Lazaridis, Iosif, Dani Nadel, Gary Rollefson, Deborah C. Merrett, Nadin Rohland, Swapan Mallick, Daniel Fernandes, et al. 2016. "Genomic Insights into the Origin of Farming in the Ancient Near East." *Nature* 536 (7617): 419–24.
Li, Heng, and Richard Durbin. 2010. "Fast and Accurate Long-Read Alignment with Burrows-Wheeler Transform." *Bioinformatics* 26 (5): 589–95.
Lipson, Mark, Anna Szécsényi-Nagy, Swapan Mallick, Annamária Pósa, Balázs Stégmár, Victoria Keerl, Nadin Rohland, Kristin Stewardson, Matthew Ferry, and Megan Michel. 2017. "Parallel Palaeogenomic Transects Reveal Complex Genetic History of Early European Farmers." *Nature* 551 (7680): 368–72.
Longin, R. 1971. "New Method of Collagen Extraction for Radiocarbon Dating." *Nature* 230 (5291): 241–42.
Lucas C, Galway-Witham J, Stringer CB, Bello SM. 2019. "Investigating the use of Paleolithic perforated batons: new evidence from Gough's Cave (Somerset, UK)." *Archaeol Anthropol Sci* 11: 5231–5255.
Mallick, Swapan, Heng Li, Mark Lipson, Iain Mathieson, Melissa Gymrek, Fernando Racimo, Mengyao Zhao, et al. 2016. "The Simons Genome Diversity Project: 300 Genomes from 142 Diverse Populations." *Nature* 538 (7624): 201–6.
Meadows, John, Vladimir Mihailovich Losovski, Olga Vladimirovna Lozovskaya, Sofya Sergeevna Chirkova, Oliver Craig, Alexandre Lucquin, and Michela Spataro. 2015. "Absolute Chronology of Upper Volga-Type Pottery: More Evidence from Zamostje 2." *Samara Journal of Science* 4 (3): 113–21.
Meadows, John, Olga Lozovskaya, Manon Bondetti, Dorothée G. Drucker, and Vyacheslav Moiseyev. 2020. "Human Palaeodiet at Zamostje 2, Central Russia: Results of Radiocarbon and Stable Isotope Analyses." *Quaternary International: The Journal of the International Union for Quaternary Research* 541: 89–103.
Meadows, John, Harry K. Robson, Daniel Groß, Charlotte Hegge, Harald Lübke, Ulrich Schmölcke, Thomas Terberger, and Bernhard Gramsch. 2018. "How Fishy Was the Inland Mesolithic? New Data from Friesack, Brandenburg, Germany." *Radiocarbon*, 1–16.
Meyer, Matthias, Qiaomei Fu, Ayinuer Aximu-Petri, Isabelle Glocke, Birgit Nickel, Juan-Luis Arsuaga, Ignacio Martínez, et al. 2014. "A Mitochondrial Genome Sequence of a Hominin from Sima de Los Huesos." *Nature* 505 (7483): 403–6.
Meyer, Matthias, and Martin Kircher. 2010. "Illumina Sequencing Library Preparation for Highly Multiplexed Target Capture and Sequencing." *Cold Spring Harbor Protocols* 2010 (6): db.prot5448.
Parry, R. F. 1929. "Recent Excavations at the Cheddar Caves." *Fortieth Annual Report of the*

Wells Natural History and Archaeological Society, for 1928, 32–36.
Patterson, Nick, Priya Moorjani, Yontao Luo, Swapan Mallick, Nadin Rohland, Yiping Zhan, Teri Genschoreck, Teresa Webster, and David Reich. 2012. "Ancient Admixture in Human History." *Genetics* 192 (3): 1065–93.
Patterson, Nick, Alkes L. Price, and David Reich. 2006. "Population Structure and Eigenanalysis." *PLoS Genetics* 2 (12): e190.
Petr, Martin, Benjamin Vernot, and Janet Kelso. n.d. "admixr—R Package for Reproducible Analyses Using ADMIXTOOLS." https://doi.org/10.1093/bioinformatics/btz030#supplementary-data.
Pettitt, P. 2003. "Discovery, nature and preliminary thoughts about Britain's first cave art." *Capra* 5. Available at: http://capra.group.shef.ac.uk/5/pettitt.pdf
Pickard, Catriona, and Clive Bonsall. 2020. "Post-Glacial Hunter-Gatherer Subsistence Patterns in Britain: Dietary Reconstruction Using FRUITS." *Archaeological and Anthropological Sciences* 12 (7): 142.
Posth, Cosimo, Gabriel Renaud, Alissa Mittnik, Dorothée G. Drucker, Hélène Rougier, Christophe Cupillard, Frédérique Valentin, et al. 2016. "Pleistocene Mitochondrial Genomes Suggest a Single Major Dispersal of Non-Africans and a Late Glacial Population Turnover in Europe." *Current Biology: CB* 26 (6): 827–33.
Price, Alkes L., Nick J. Patterson, Robert M. Plenge, Michael E. Weinblatt, Nancy A. Shadick, and David Reich. 2006. "Principal Components Analysis Corrects for Stratification in Genome-Wide Association Studies." *Nature Genetics* 38 (8): 904–9.
Prüfer, Kay, Fernando Racimo, Nick Patterson, Flora Jay, Sriram Sankararaman, Susanna Sawyer, Anja Heinze, et al. 2014. "The Complete Genome Sequence of a Neanderthal from the Altai Mountains." *Nature* 505 (7481): 43–49.
Quinlan, Aaron R., and Ira M. Hall. 2010. "BEDTools: A Flexible Suite of Utilities for Comparing Genomic Features." *Bioinformatics* 26 (6): 841–42.
Raghavan, Maanasa, Pontus Skoglund, Kelly E. Graf, Mait Metspalu, Anders Albrechtsen, Ida Moltke, Simon Rasmussen, et al. 2014. "Upper Palaeolithic Siberian Genome Reveals Dual Ancestry of Native Americans." *Nature* 505 (7481): 87–91.
Reade, Hazel, Jennifer A. Tripp, Sophy Charlton, Sonja Grimm, Kerry L. Sayle, Alex Fensome, Thomas F. G. Higham, Ian Barnes, and Rhiannon E. Stevens. 2020. "Radiocarbon Chronology and Environmental Context of Last Glacial Maximum Human Occupation in Switzerland." *Scientific Reports* 10 (1): 4694.
Rees, Catherine, and George Nash. 2017. "Recent Archaeological Investigations at Kendrick's Upper Cave, Great Orme, Llandudno." *Proceedings of the University of Bristol Spelaeological Society* 27 (2): 185–96.
Reimer, Paula J., William E. N. Austin, Edouard Bard, Alex Bayliss, Paul G. Blackwell, Christopher Bronk Ramsey, Martin Butzin, et al. 2020. "The IntCal20 Northern Hemisphere Radiocarbon Age Calibration Curve (0–55 Cal kBP)." *Radiocarbon* 62 (4): 725–57.
Renaud, Gabriel, Viviane Slon, Ana T. Duggan, and Janet Kelso. 2015. "Schmutzi: Estimation of Contamination and Endogenous Mitochondrial Consensus Calling for Ancient DNA." *Genome Biology* 16 (October): 224.
Richards, M. P., R. E. M. Hedges, R. Jacobi, A. Current, and C. Stringer. 2000. "FOCUS: Gough's Cave and Sun Hole Cave Human Stable Isotope Values Indicate a High Animal Protein Diet in the British Upper Palaeolithic." *Journal of Archaeological Science* 27 (1): 1–3.
Richards, M. P., R. Jacobi, J. Cook, P. B. Pettitt, and C. B. Stringer. 2005. "Isotope Evidence for the Intensive Use of Marine Foods by Late Upper Palaeolithic Humans." *Journal of Human Evolution* 49 (3): 390–94.
Rohland, Nadin, Eadaoin Harney, Swapan Mallick, Susanne Nordenfelt, and David Reich. 2015. "Partial uracil–DNA–glycosylase Treatment for Screening of Ancient DNA." *Philosophical*

Transactions of the Royal Society of London. Series B, Biological Sciences 370 (1660): 20130624.
Sayle, Kerry L., Christopher R. Brodie, Gordon T. Cook, and W. Derek Hamilton. 2019. "Sequential Measurement of δ15 N, δ13 C and δ34 S Values in Archaeological Bone Collagen at the Scottish Universities Environmental Research Centre (SUERC): A New Analytical Frontier." *Rapid Communications in Mass Spectrometry: RCM* 33 (15): 1258–66.
Shennan, Ian, Sarah Bradley, Glenn Milne, Anthony Brooks, Sophie Bassett, and Sarah Hamilton. 2006. "Relative Sea-Level Changes, Glacial Isostatic Modelling and Ice-Sheet Reconstructions from the British Isles since the Last Glacial Maximum." *Journal of Quaternary Science* 21 (6): 585–99.
Sieveking, G. de G. 1971. "The Kendrick's Cave Mandible." *The British Museum Quarterly* 35 (1/4): 230–50.
Skoglund, Pontus, Bernd H. Northoff, Michael V. Shunkov, Anatoli P. Derevianko, Svante Pääbo, Johannes Krause, and Mattias Jakobsson. 2014. "Separating Endogenous Ancient DNA from Modern Day Contamination in a Siberian Neandertal." *Proceedings of the National Academy of Sciences of the United States of America* 111 (6): 2229–34.
Skoglund, Pontus, Jan Storå, Anders Götherström, and Mattias Jakobsson. 2013. "Accurate Sex Identification of Ancient Human Remains Using DNA Shotgun Sequencing." *Journal of Archaeological Science* 40 (12): 4477–82.
Skoglund, Pontus, Jessica C. Thompson, Mary E. Prendergast, Alissa Mittnik, Kendra Sirak, Mateja Hajdinjak, Tasneem Salie, et al. 2017. "Reconstructing Prehistoric African Population Structure." *Cell* 171 (1): 59–71.e21.
Stevens, Rhiannon E., and Robert E. M. Hedges. 2004. "Carbon and Nitrogen Stable Isotope Analysis of Northwest European Horse Bone and Tooth Collagen, 40,000 BP–present: Palaeoclimatic Interpretations." *Quaternary Science Reviews* 23 (7–8): 977–91.
Stevens, Rhiannon E., Roger M. Jacobi, and Thomas F. G. Higham. 2010/1. "Reassessing the Diet of Upper Palaeolithic Humans from Gough's Cave and Sun Hole, Cheddar Gorge, Somerset, UK." *Journal of Archaeological Science* 37 (1): 52–61.
Stringer, C. 2000. "The Gough's Cave Human Fossils: An Introduction." *BULLETIN-NATURAL HISTORY MUSEUM GEOLOGY SERIES* 56 (2): 135–40.
Szpak, Paul, Jessica Z. Metcalfe, and Rebecca A. Macdonald. 2017. "Best Practices for Calibrating and Reporting Stable Isotope Measurements in Archaeology." *Journal of Archaeological Science: Reports* 13 (June): 609–16.
Villalba-Mouco, Vanessa, Marieke S. van de Loosdrecht, Cosimo Posth, Rafael Mora, Jorge Martínez-Moreno, Manuel Rojo-Guerra, Domingo C. Salazar-García, José I. Royo-Guillén, Michael Kunst, and Hélène Rougier. 2019. "Survival of Late Pleistocene Hunter-Gatherer Ancestry in the Iberian Peninsula." *Current Biology: CB* 29 (7): 1169–77. e7.
