# Supplementary material for: Dual ancestries and ecologies of the Late Glacial Palaeolithic in Britain
Source: Nat Ecol Evol. 2022 Oct 24;6(11):1658–68. doi: 10.1038/s41559-022-01883-z (PMC9630104; doi:10.1038/s41559-022-01883-z)
Supplement: Supplementary file 2 — Reporting Summary [file 41559_2022_1883_MOESM2_ESM.pdf]

nature portfolio

Corresponding author(s): Sophy Charlton

Last updated by author(s): 9 December, 2021

Reporting Summary

Nature Portfolio wishes to improve the reproducibility of the work that we publish. This form provides structure for consistency and transparency in reporting. For further information on Nature Portfolio policies, see our Editorial Policies and the Editorial Policy Checklist.

Statistics

For all statistical analyses, confirm that the following items are present in the figure legend, table legend, main text, or Methods section.

n/a	Confirmed	
☐	☒	The exact sample size (*n*) for each experimental group/condition, given as a discrete number and unit of measurement
☐	☒	A statement on whether measurements were taken from distinct samples or whether the same sample was measured repeatedly
☐	☒	The statistical test(s) used AND whether they are one- or two-sided<br>*Only common tests should be described solely by name; describe more complex techniques in the Methods section.*
☐	☒	A description of all covariates tested
☐	☒	A description of any assumptions or corrections, such as tests of normality and adjustment for multiple comparisons
☐	☒	A full description of the statistical parameters including central tendency (e.g. means) or other basic estimates (e.g. regression coefficient) AND variation (e.g. standard deviation) or associated estimates of uncertainty (e.g. confidence intervals)
☐	☒	For null hypothesis testing, the test statistic (e.g. *F*, *t*, *r*) with confidence intervals, effect sizes, degrees of freedom and *P* value noted<br>*Give P values as exact values whenever suitable.*
☐	☒	For Bayesian analysis, information on the choice of priors and Markov chain Monte Carlo settings
☐	☒	For hierarchical and complex designs, identification of the appropriate level for tests and full reporting of outcomes
☒	☐	Estimates of effect sizes (e.g. Cohen's *d*, Pearson's *r*), indicating how they were calculated

Our web collection on statistics for biologists contains articles on many of the points above.

Software and code

Policy information about availability of computer code

Data collection	No code or software was used in data collection
Data analysis	For the aDNA analysis, the following software was used: PMDtools (Skoglund et al. 2014), sex identification using the Python script in Skoglund et al. 2013, Burrows Wheeler Aligner (BWA) (Li and Durbin 2010), schmutzi (Renaud et al. 2015), HaploGrep (Kloss-Brandstätter et al. 2011), MAFFT v7.271 (Katoh and Standley 2013), FigTree (version: v1.4.2) (http://tree.bio.ed.ac.uk/software/figtree/), BEDtools (version 2.23.0) (Quinlan and Hall 2010), smartpca from the EIGENSOFT package (Patterson, Price and Reich 2006; Price et al. 2006), ADMIXTOOLS (version 5.1) (Patterson et al. 2012), and the R package admixr (version 0.9.1) (Petr, Vernot, and Kelso, n.d.).<br>For the AMS analysis, the following software was used: OxCal (version 4.4) (Bronk Ramsey 2021) and IntCal20 (Reimer et al. 2020). The CQL code for the OxCal model used in the manuscript is given in the Supplementary Information, and the output can be found in Supplementary File 5.<br>For the isotopic analysis, the following software was used: FRUITS (Fernandes et al. 2014)

For manuscripts utilizing custom algorithms or software that are central to the research but not yet described in published literature, software must be made available to editors and reviewers. We strongly encourage code deposition in a community repository (e.g. GitHub). See the Nature Portfolio guidelines for submitting code & software for further information.

Data

Policy information about availability of data

All manuscripts must include a data availability statement. This statement should provide the following information, where applicable:

- Accession codes, unique identifiers, or web links for publicly available datasets
- A description of any restrictions on data availability
- For clinical datasets or third party data, please ensure that the statement adheres to our policy

BAM files (one file per library) have been deposited in the European Nucleotide Archive under study accession number PRJEB52727. Stable isotope data generated in this study can be found in Supplementary File 3. The CQL code for the OxCal model used in the manuscript is given in the Supplementary Information, and the output can be found in Supplementary File 5.

Field-specific reporting

Please select the one below that is the best fit for your research. If you are not sure, read the appropriate sections before making your selection.

☐ Life sciences ☐ Behavioural & social sciences ☒ Ecological, evolutionary & environmental sciences

For a reference copy of the document with all sections, see nature.com/documents/nr-reporting-summary-flat.pdf

Ecological, evolutionary & environmental sciences study design

All studies must disclose on these points even when the disclosure is negative.

Study description	A multi-methodological biomolecular study of human skeletal material from two Late Glacial Palaeolithic sites in Britain. The study utilises ancient DNA analysis, AMS dating and stable isotopic analyses.
Research sample	From Gough's Cave, one human temporal bone (PV M 96544 (excavation numbers GC 86 (55) and GC 87 (60)) was uitlised for aDNA analysis. From Kendrick's Cave, four human skeletal elements were re-AMS dated, and one human mandibular first molar (M1) (Kendricks_074) was also targeted for aDNA analysis.
Sampling strategy	One individual from each site was chosen for aDNA analysis. Human skeletal material from Late Pleistocene Britain is extremely rare, and to date, modern human skeletal remains have been recovered from only six Upper Palaeolithic sites in the UK. However, these rare samples are crucial for our understanding of human populations across post-LGM Europe due to Britain’s location on the most northwesterly fringe of the European continent. Four human skeletal elements from Kendrick's Cave were also sampled for AMS as although there are existing dates from the site, only one of these previous dates included ultrafiltration in the pretreatment procedure. Furthermore, the diet of individuals at the site has not previously been considered when calibrating these dates, which is necessary due to a marine and/or freshwater component in their diet. Therefore we re-dated four of the human bones and incorporated dietary information into the radiocarbon calibration.
Data collection	aDNA laboratory protocols (ancient DNA extraction and library preparation) were undertaken in the dedicated ancient DNA laboratory at the Natural History Museum, London. Libraries were sequenced at The Francis Crick Institute, London. AMS dating of the Kendrick’s Cave material was undertaken at the Oxford Radiocarbon Accelerator Unit (ORAU), following sample preparation at University College London (UCL). Collagen extraction for isotopic analyses was also undertaken at University College London (UCL).
Timing and spatial scale	N/A
Data exclusions	No data was excluded
Reproducibility	N/A
Randomization	N/A
Blinding	N/A

Did the study involve field work? ☐ Yes ☒ No

Reporting for specific materials, systems and methods

We require information from authors about some types of materials, experimental systems and methods used in many studies. Here, indicate whether each material, system or method listed is relevant to your study. If you are not sure if a list item applies to your research, read the appropriate section before selecting a response.

Materials & experimental systems

n/a	Involved in the study
☒	☐ Antibodies
☒	☐ Eukaryotic cell lines
☐	☒ Palaeontology and archaeology
☒	☐ Animals and other organisms
☒	☐ Human research participants
☒	☐ Clinical data
☒	☐ Dual use research of concern

Methods

n/a	Involved in the study
☒	☐ ChIP-seq
☒	☐ Flow cytometry
☒	☐ MRI-based neuroimaging

Palaeontology and Archaeology

Specimen provenance

Permission to sample the Kendrick's Cave material was granted by Conwy County Borough Council and Llandudno Museum. Access and permissions to sample the Gough's Cave material were granted by the Longleat Estate, the Natural History Museum London, and Dr Heather Bonney (Principal Curator, Human Remains and Anthropology, NHM).

Specimen deposition

The material utilised in the study is held by Llandudno Museum and the Natural History Museum London.

Dating methods

Four new AMS dates were obtained from four skeletal elements from the Kendrick's Cave assemblage. Samples were prepared at University College London (UCL) using a modified version of the Oxford Radiocarbon Accelerator Unit (ORAU) collagen extraction procedure (Brock et al. 2010), which is based on a modified version of the (Longin 1971) protocol. Full details of this are provided in the Supplementary Information. Samples were analysed using a Delta V Advantage continuous-flow isotope ratio mass spectrometer coupled via a ConfloIV to an EA IsoLink elemental analyser (Thermo Fisher Scientific, Bremen) at the Scottish Universities Environmental Research Centre (SUERC). Measurement uncertainty was determined to be ±0.1‰ for δ13C and ±0.2‰ for δ15N on the basis of repeated measurements of an in-house bone collagen standard and a certified fish gelatin standard (Elemental Microanalysis, UK). Each sample was analysed in duplicate with the exception of one sample (UPN-643, Museum No 069) and reproducibility was better than ±0.1‰ for δ13C and ±0.2‰ for δ15N. Results for this isotopic work are given in Supplementary File 3. Dates on samples prepared in the laboratory at UCL were further corrected for laboratory background carbon as outlined in (Reade et al. 2020). Corrected dates are indicated by a "C". Dates were calibrated and modelled using the Bayesian statistical program OxCal (version 4.4) (Bronk Ramsey 2021), applying the IntCal20 calibration curve (Reimer et al. 2020).

☒ Tick this box to confirm that the raw and calibrated dates are available in the paper or in Supplementary Information.

Ethics oversight

No ethical approval was required as the material utilised in this study does not fall under the Human Tissue Act 2004.

Note that full information on the approval of the study protocol must also be provided in the manuscript.
